# Ligand-binding and -scavenging of the chemerin receptor GPR1

**DOI:** 10.1007/s00018-021-03894-8

**Published:** 2021-07-09

**Authors:** Tobias F. Fischer, Anne S. Czerniak, Tina Weiß, Clara T. Schoeder, Philipp Wolf, Oliver Seitz, Jens Meiler, Annette G. Beck-Sickinger

**Affiliations:** 1 Institute of Biochemistry, Leipzig University, Brüderstraße 34, 04103 Leipzig, Germany; 2 Center for Structural Biology, Department of Chemistry, Vanderbilt University, 465 21st Avenue South, Nashville, TN37212 USA; 3 Department of Chemistry, Humboldt-Universität Zu Berlin, Brook-Taylor-Str. 2, 12489 Berlin, Germany; 4 Institute for Drug Discovery, Leipzig University Medical School, 04103 Leipzig, Germany

**Keywords:** Adipokine, Inflammation, GPCR, Atypical chemokine receptor

## Abstract

Tight regulation of cytokines is essential for the initiation and resolution of inflammation. Chemerin, a mediator of innate immunity, mainly acts on chemokine-like receptor 1 (CMKLR1) to induce the migration of macrophages and dendritic cells. The role of the second chemerin receptor, G protein-coupled receptor 1 (GPR1), is still unclear. Here we demonstrate that GPR1 shows ligand-induced arrestin3 recruitment and internalization. The chemerin C-terminus triggers this activation by folding into a loop structure, binding to aromatic residues in the extracellular loops of GPR1. While this overall binding mode is shared between GPR1 and CMKLR1, differences in their respective extracellular loop 2 allowed for the design of the first GPR1-selective peptide. However, our results suggest that ligand-induced arrestin recruitment is not the only mode of action of GPR1. This receptor also displays constitutive internalization, which allows GPR1 to internalize inactive peptides efficiently by an activation-independent pathway. Our results demonstrate that GPR1 takes a dual role in regulating chemerin activity: as a signaling receptor for arrestin-based signaling on one hand, and as a scavenging receptor with broader ligand specificity on the other.

**Graphic abstract:**

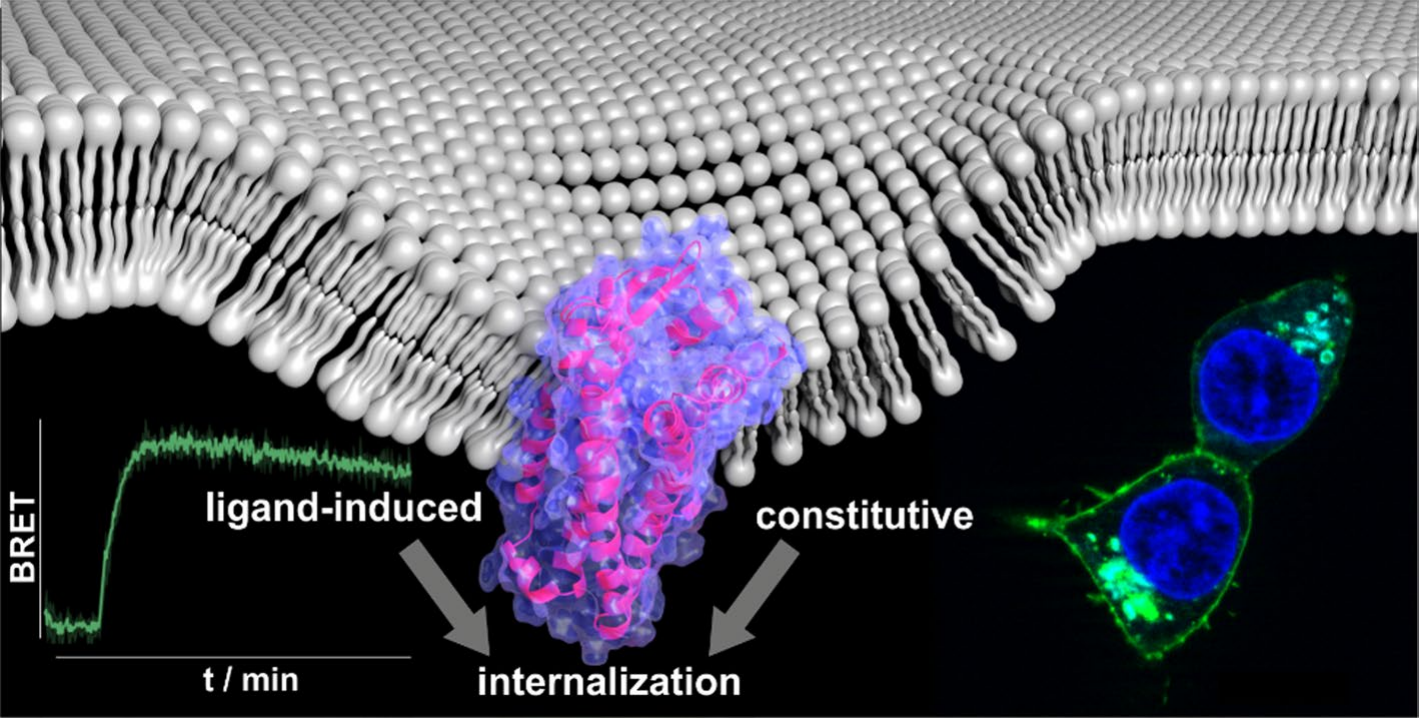

**Supplementary Information:**

The online version contains supplementary material available at 10.1007/s00018-021-03894-8.

## Introduction

G protein-coupled receptor 1 (GPR1) is a G protein-coupled receptor (GPCR) that was first identified as an orphan receptor in the human hippocampus in 1994 [1]. It was not until 14 years later that Barnea et al. reported chemerin as a natural ligand for GPR1 in 2008 [2]. Chemerin is a small protein that is expressed in adipose tissue, liver, and skin [3, 4]. After cleavage of an N-terminal signal peptide, it is secreted as 143 amino acid prochemerin, [5] followed by activation through C-terminal processing by proteases of the coagulation and inflammatory cascades [6, 7]. The resulting chemerin biologically active isoforms are named according to their last C-terminal amino acid, ChemS157 (consisting of 137 residues) and ChemF156 (consisting of 136 residues) [8]. Chemerin is predicted to form a cystatin-like fold, thus sharing structural homology with human cathelidin [9, 10]. Levels of chemerin strongly correlate with the body mass index (BMI) and further obesity-associated parameters, such as fasting serum insulin or hypertension [11, 12]. Moreover, chemerin is linked to many co-morbidities of obesity, such as metabolic syndrome, psoriasis, or diabetes [13]. The role of chemerin in auto-inflammatory diseases has gained increasing interest: after it was initially isolated from the synovial joint fluid from patients suffering from rheumatoid arthritis, there is emerging evidence that chemerin is a driver of inflammation in the joints of these individuals [5, 14]. Chemerin levels are also elevated in early psoriatic skin lesions, where it correlates with infiltration by dendritic cells [15]. More recently, several studies have demonstrated that chemerin plays essential roles in different cancer types: either indirectly by promoting angiogenesis in, e.g., colorectal cancer, [16] or directly by stimulating the invasion of oesophageal squamous cancer cells [17].

Chemerin binds to three GPCRs with different affinities: GPR1, chemokine-like receptor 1 (CMKLR1), and C–C motif chemokine receptor-like 2 (CCRL2). While the highest affinity was found for GPR1 [18], most known functions are mediated by CMKLR1, e.g., the chemotaxis of leukocytes towards sites of inflammation, the differentiation of adipocytes, and vasoconstrictive effects of chemerin [5, 19, 20]. In contrast, the biological role of GPR1 is understudied. GPR1 knock-out mice displayed exacerbated glucose intolerance after being fed with a high-fat diet [21]. Other studies suggest a regulatory role of GPR1 in follicle development and hormone secretion [22]. No GPR1-selective probe molecules are known so far, which impedes the characterization of this receptor [23].

GPR1 shares high sequence identity (35.1%) with CMKLR1, its closest homolog, which is expressed by adipocytes and cells of the innate immune system [4, 5, 24]. However, while CMKLR1 acts as a classical GPCR with functional homology to the chemokine receptors, no ligand-induced G protein activation has been described for GPR1 [18]. The functional response induced by GPR1 upon stimulation with chemerin is limited to arrestin recruitment and RhoA/ROCK-mediated signaling [2, 25]. For the third chemerin receptor, CCRL2, no detectable signaling events have been observed [18, 26].

The identification and characterization of atypical chemokine receptors was a significant step in the characterization of the chemokine system. The chemerin system displays many parallels to the chemokines, and insights into the mechanisms that control and mediate chemerin activity will be essential to understand the chemerin system. We, therefore, chose to analyze the function of GPR1. The determination of the ligand-binding mode of chemerin at GPR1 enabled us to develop the first selective ligand for this receptor. Moreover, we demonstrate that GPR1 can scavenge and internalize peptides that fail to induce receptor activation.

## Results

**Fig. 1:**
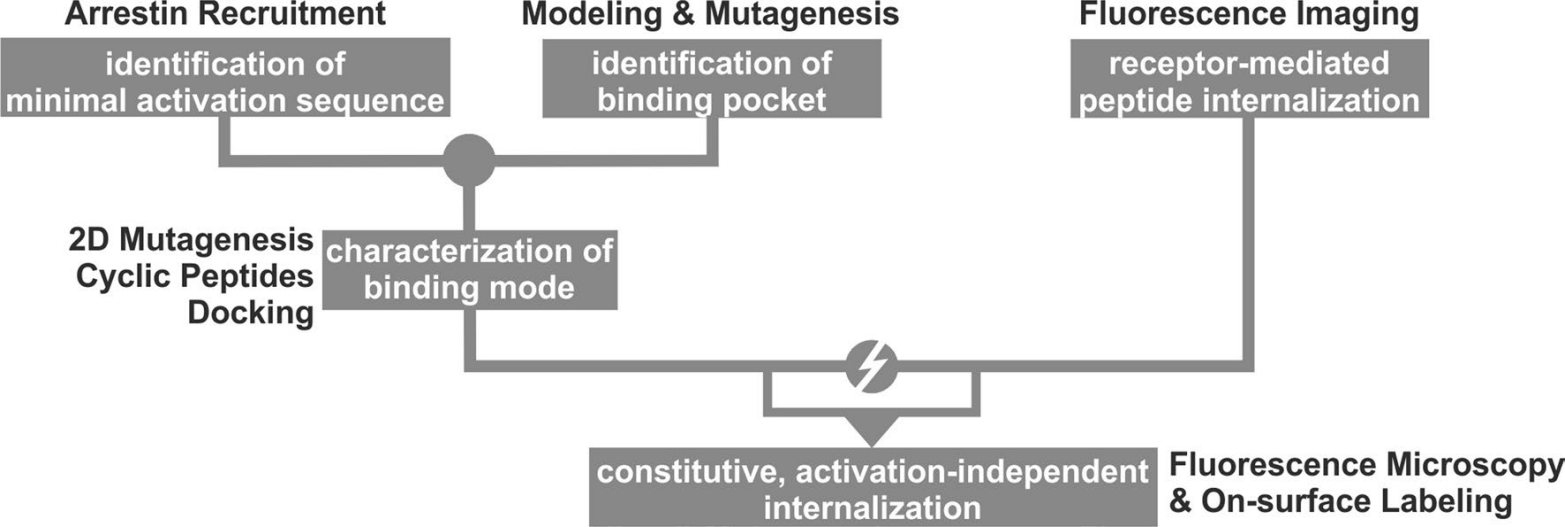
Flow chart of the experimental strategy to characterize the chemerin receptor GPR1 combining experimental and computational techniques

We devised an experimental/computational protocol outlined in Fig. 1 to study the structure–function relation of chemerin with its receptor GPR1. First, we determined the minimal portion of the chemerin protein needed for full arrestin recruitment, our functional readout, to identify the portion of chemerin that engages the receptor. In parallel, we constructed a comparative model of GPR1 to identify candidate residues in the extracellular loops and the upper portions of the transmembrane (TM) helices that can form the putative-binding site for chemerin; this was done bearing in mind the previously identified binding pocket of chemerin at the CMKLR1 [27]. Consequently, we determined an interaction point by complementary mutagenesis, and probed the internal conformation of the minimal activation sequence employing cyclized peptides. Using our experimental data as restraints, we docked the minimal peptide into the GPR1-binding pocket. As our results from the studies of the binding mode based on arrestin recruitment as a readout were not in agreement with results obtained in fluorescence microscopy, we further investigated the internalization of GPR1 and its ligands, revealing an activation-independent pathway of constitutive internalization.

### A C-terminal chemerin peptide is sufficient to induce rapid recruitment of arrestin to GPR1

**Fig. 2:**
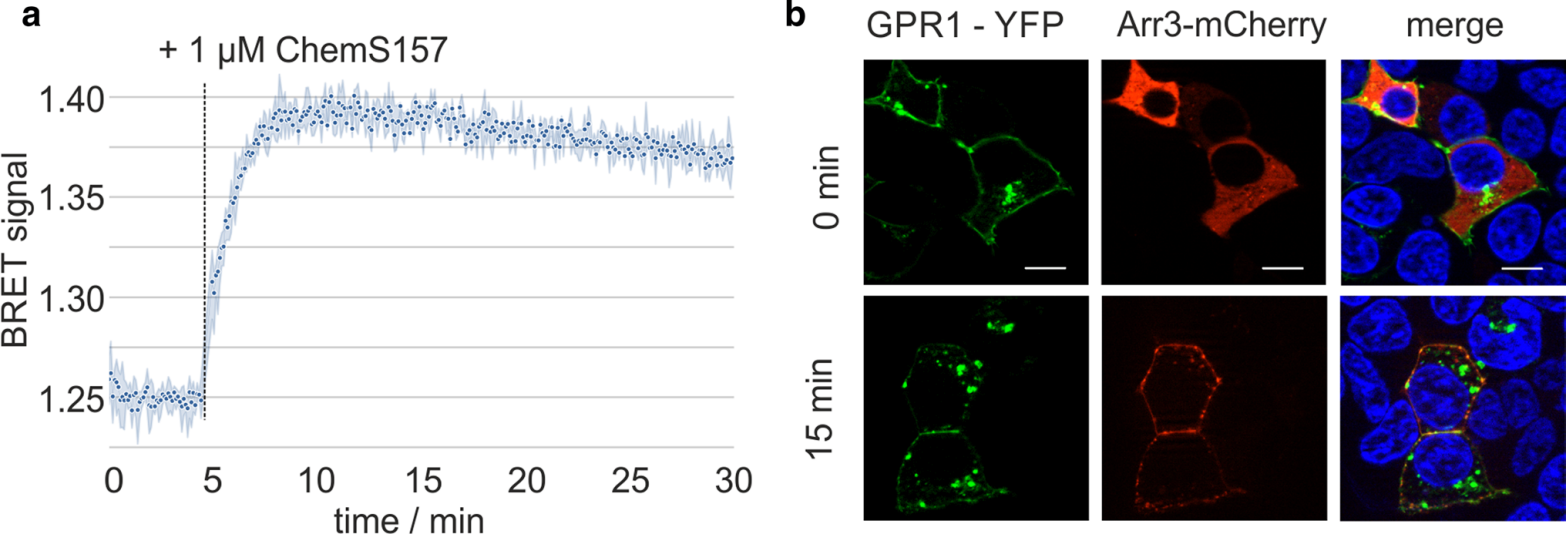
GPR1 rapidly recruits arrestin 3 and internalizes upon ligand stimulation. **a** Recruitment of Rluc8-Arr3 by GPR1 in response to stimulation with full-length ChemS157 over time, data points represent mean of three technical replicates with shaded 95% confidence interval (CI). **b** GPR1-eYFP and Arr3-mCherry, before and 15 min after stimulation with 1 µM ChemS157. GPR1 internalizes without arrestin3, which stays at the membrane. Cell nuclei were stained with Hoechst 33342; scale bar = 10 µm

Stimulation with ChemS157 led to rapid recruitment of arrestin3 to the receptor, reaching its maximum within 2 min as monitored by a bioluminescence resonance energy transfer (BRET) assay (Fig. 2a). Fluorescence microscopy showed that GPR1 recruits mCherry-labeled arrestin3 to the membrane upon stimulation with 1 µM ChemS157. However, GPR1 internalizes without arrestin3, leaving Arr3-mCherry at the membrane (Fig. 2b).

To further characterize the activation of GPR1 by chemerin, we tested the effect of truncations of the ligand on arrestin3-recruitment in a BRET assay. Recombinantly expressed ChemS157 displayed a low nanomolar activity (EC_50_ = 2.1 nM). The same activity was observed for recombinantly expressed ChemF156 (EC_50_ = 2.6 nM). Next, we synthesized two peptides derived from the C-termini of ChemS157 and ChemF156, Chem^139−157^ and Chem^139−156^. These peptides displayed the same activities as the full-length proteins with EC_50_ values of 3.1 nM and 2.9 nM, respectively. Further truncating the peptide to yield Chem^149−157^ (chemerin-9) and Chem^149−156^ had no negative impact on activity. However, further N-terminal truncations resulted in a loss of activity: Chem^150−157^ was tenfold less active than the full-length protein, while Chem^151−157^ was completely devoid of activity at concentrations up to 1 µM. Similarly, removal of the C-terminal Phe^156^ resulted in a complete loss of activity; Chem^149−155^ (chemerin-7) did not reach full receptor activation at concentrations of up to 1 µM. Two scrambled chemerin-9 peptides (scrC9 and scr2C9) failed to induce arrestin3-recruitment at concentrations of up to 10 µM. Table 1 displays an overview of all EC_50_ values, BRET profiles of all peptides are displayed in Figure S1.

**Table 1:** Activity of chemerin and derived proteins and peptides at the GPR1 in a BRET-based arrestin3-recruitment assay. Nonlinear regression was performed in GraphPad Prism 5, with mean values from at least two independent experiments performed in quadruplicates

Peptide	Sequence	EC_50_/nM	pEC_50_ ± SEM	E_max_/%
ChemS157	21–157	2.1	8.68 ± 0.14	103 ± 8
ChemF156	21–156	2.6	8.57 ± 0.16	106 ± 9
Chem^139−157^	QRAGEDPHSFYFPGQFAFS	3.1	8.51 ± 0.14	94 ± 7
Chem^139−156^	QRAGEDPHSFYFPGQFAF	2.9	8.53 ± 0.17	99 ± 9
Chem^149−157^ (= chemerin-9)	YFPGQFAFS	1.9	8.76 ± 0.09	101 ± 4
Chem^150−157^	FPGQFAFS	22	7.65 ± 0.16	87 ± 8
Chem^151−157^	PGQFAFS	> 1,000	< 6	n.d
Chem^149−156^	YFPGQFAF	4.0	8.40 ± 0.34	110 ± 16
Chem^149−155^ (chemerin-7)	YFPGQFA	> 1,000	< 6	n.d
scrC9	GYFPFQASF	> 1,000	< 6	n.d
scr2C9	QFYSFFPAG	> 1,000	< 6	n.d
[L^8^] chemerin-9	YFPGQFALS	1.4	8.86 ± 0.19	125 ± 12

### Identification of a putative chemerin-9 binding pocket through homology modeling

**Fig. 3:**
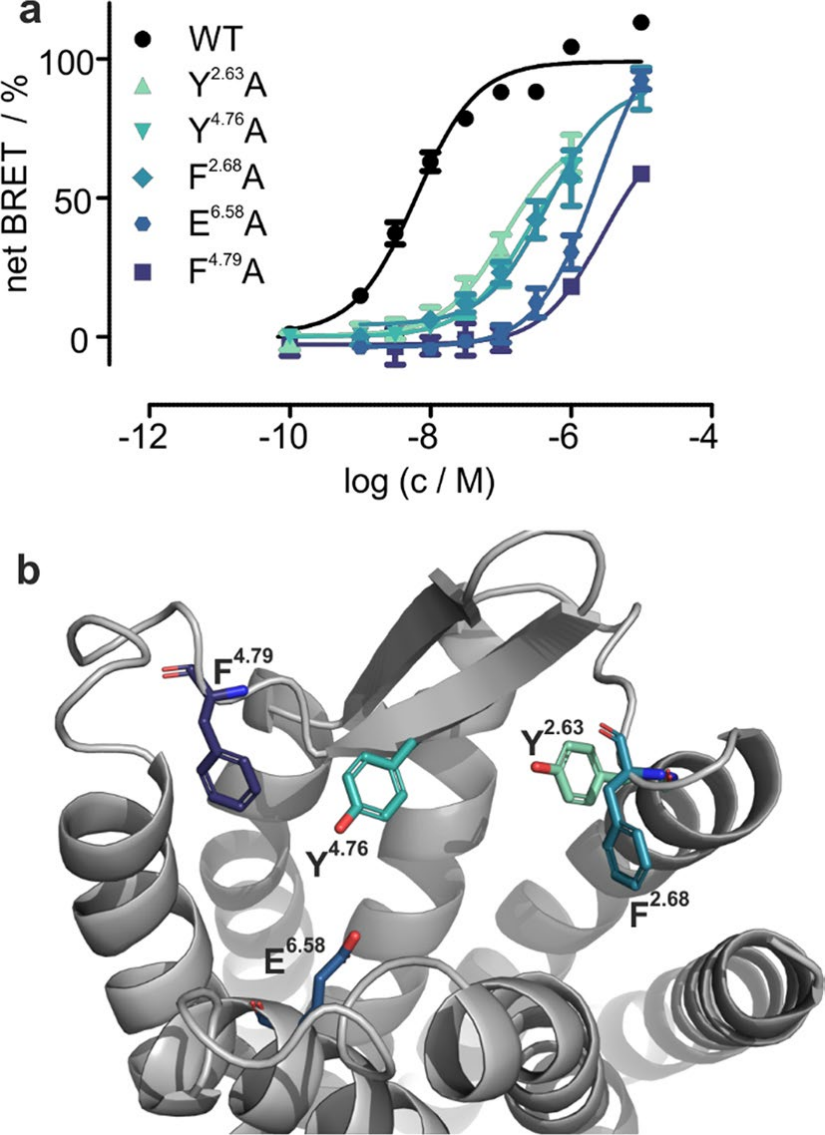
Identification of the ligand-binding pocket at GPR1. **a** Mutagenesis data obtained in a nanoBRET-binding assay revealed several conserved, mostly aromatic residues in the extracellular loops and upper TMs to be involved in ligand binding. Concentration–response curves of Nluc-tagged GPR1 and mutants stimulated with Tam-EG(4)-chemerin-9 are shown. Data points represent mean ± SEM from at least two independent experiments performed in triplicates. **b** Close-up view of the binding pocket in the best scoring GPR1 model with experimentally confirmed key residues shown as sticks

For an improved understanding of the function of GPR1, knowledge of the peptide-binding mode is essential. To gain insight into the three-dimensional structure of the receptor, we constructed homology models of GPR1 employing RosettaCM. We chose crystal structures of five related receptors as templates for homology modeling: complement 5 a receptor 1 (PDB: 6c1r), type 1 angiotensin II receptor (AT1R, PDB: 4zud), apelin receptor (APJ, PDB: 5vbl), CXC chemokine receptor 4 (CXCR4, PDB: 3odu) and CC chemokine receptor 9 (CCR9, PDB: 5lwe), which display sequence identities of 27–35% to GPR1. In a previous benchmark of our method, these premises proved suitable for constructing highly accurate homology models from multiple templates [28]. Details on the construction of homology models are given in the methods section and SI. As the receptor N- and C-termini are expected to be highly flexible, they were truncated to limit the conformational sampling space. A total of 1500 models were produced and clustered by Cα RMSD (Figure S2), the binding pocket of the best scoring model is displayed in Fig. 3b. We selected conserved residues in the extracellular loops of GPR1 that pointed to the putative-binding pocket for investigation in a nanoBRET-based ligand-binding assay: residues Y^2.63^, F^2.68^, Y^4.76^, and F^4.79^ (numbering of receptor residues follows Ballesteros and Weinstein [29]) are highly conserved across species and potentially available for interaction with the aromatic residues in the peptide. Residue E^6.58^ is positioned as in CMKLR1, where it is critical for binding to chemerin-9, suggesting a similar role in GPR1 [27].

### Residues in the extracellular loops of GPR1 interact with chemerin-9

Mutations were introduced into a GPR1 construct N-terminally fused to NanoLuc®. The receptor was stimulated with chemerin-9 N-terminally connected to a 6-carboxy tetramethylrhodamine (Tam) fluorophore by an ethylene glycol linker (Tam-EG(4)-chemerin-9). Stimulating Nluc-GPR1 WT with Tam-EG(4)-chemerin-9 yielded a nanomolar affinity (EC_50_ = 6 nM). Exchanging residues Y^2.63^ and F^2.68^ in TM2 and ECL1 to alanine led to a pronounced loss of affinity with EC_50_ values of 114 nM and 460 nM, respectively. Two residues in the ECL2, Y^4.76^, and F^4.79^, also displayed a dramatic loss of affinity when exchanged to alanine (EC_50_ = 224 nM and EC_50_ > 1000 nM, respectively). E^6.58^ was the only non-aromatic residue we found to be essential for peptide binding; the E^6.58^A mutant displayed an EC_50_ > 1000 nM.

### A hydrophobic pocket formed by the ECL2 of GPR1 contributes to ligand binding

**Fig. 4:**
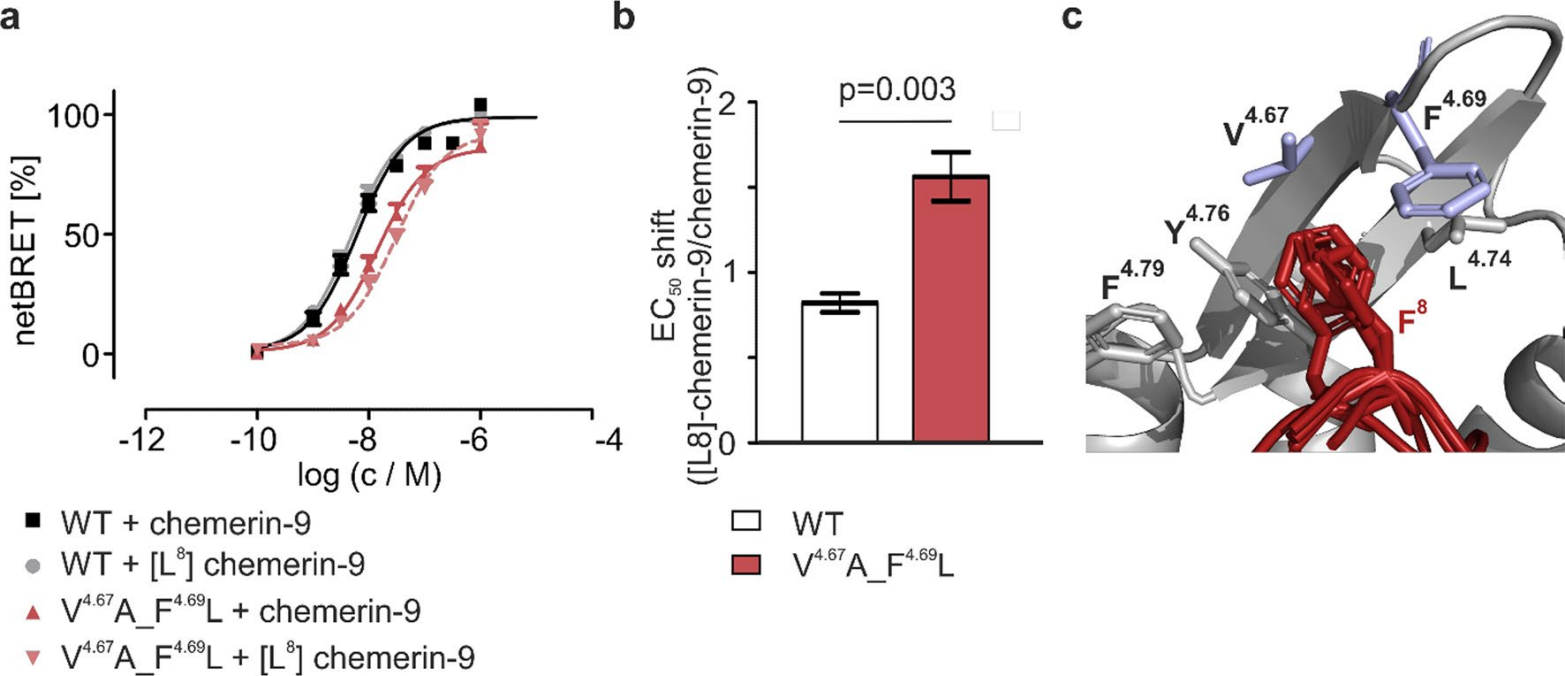
Chemerin-9 residue F^8^ interacts with a hydrophobic pocket in the ECL2 of GPR1. **a** Stimulating GPR1 with [L^8^]-chemerin-9 yields no shift in affinity compared to the wild-type peptide, because the bulky residues in ECL2 can compensate for this modification. At the V^4.67^A_F^4.69^L mutant, stimulation with [L^8^]-chemerin-9 shows a slight shift compared to stimulation with the wild-type peptide, possibly because this mutant has a wider ECL2. Binding data were obtained by stimulating the Nluc-tagged GPR1 variant with N-terminally Tam-labeled peptides. Data points are mean ± SEM from four independent experiments performed in triplicates. **b** Shift at the V^4.67^A_F^4.69^L mutant is significantly bigger than at the WT receptor. Mean values with their 95% CI are displayed. Statistical significance was tested with an unpaired, two-tailed *t* test. **c** Close-up of the binding site in the five best scoring docked models with the ligand shown in red, chemerin-9 residue F^8^ shown as sticks. Receptor residues forming the hydrophobic pocket in ECL2 are displayed as sticks, the mutated residues V^4.67^ and F^4.69^ are highlighted in blue

Chemerin-9 residue F^8^ binds to a hydrophobic pocket in the ECL2 of CMKLR1 [27]. Most of the ligand-binding residues are conserved between CMKLR1 and GPR1. We, therefore, investigated whether an interaction between chemerin-9 residue F^8^ and the ECL2 of GPR1 is involved in ligand binding as well. Introducing the F^8^L mutation in the ligand ([L^8^]-chemerin-9) resulted in a very slight increase of affinity at the wild-type receptor (EC_50_ = 4.8 nM), representing a 0.8-fold shift of affinity (95% CI 0.71–0.93). Replacing V^4.67^ and F^4.69^ with sterically less demanding A and L residues, respectively (V^4.67^A_F^4.69^L), resulted in a decreased affinity (EC_50_ = 14 nM). At the V^4.67^A_F^4.69^L mutant, [L^8^]-chemerin-9 displayed a further decreased activity (EC_50_ = 22 nM) corresponding to a 1.5-fold loss of affinity (95% CI 1.3–1.8). These results demonstrate that the wild-type receptor can fully compensate for smaller ligand residues binding to ECL2, while the pocket of the V^4.67^A_F^4.69^L mutant, containing smaller side chains, is less suitable to do so Fig. 4.

**Fig. 5:**
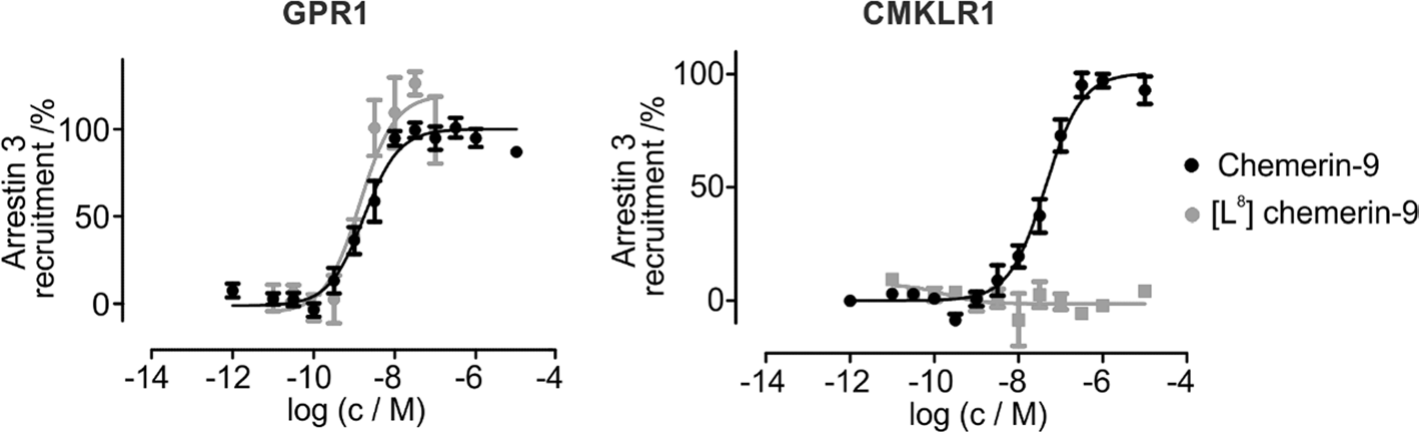
[L^8^]-chemerin-9 is a selective ligand for GPR1 in arrestin3 recruitment assays. Stimulating GPR1 with chemerin-9 demonstrates a high potency of the peptide to recruit arrestin3 in a BRET assay (EC_50_ = 1.9 nM). [L^8^]-chemerin-9 displays identical activity (EC_50_ = 1.4 nM). Stimulating CMKLR1 with increasing concentrations of chemerin-9 displays nanomolar activity of the peptide (EC_50_ = 44 nM), [L^8^]-chemerin-9 does not trigger recruitment of arrestin3 to the receptor. Data points represent mean ± SEM from at least two independent experiments performed in quadruplicates. Data were normalized to the top and bottom values of the respective chemerin-9 curve

The hydrophobic pocket formed by the ECL2 of CMKLR1 resembles that of the GPR1 V^4.67^A_F^4.69^L mutant. Therefore, we tested whether [L^8^]-chemerin-9 shows selectivity for GPR1 over CMKLR1 in a BRET-based arrestin3-recruitment assay (Fig. 5). Indeed, [L^8^]-chemerin-9 displays a high activity at GPR1 (EC_50_ = 1.8 nM). While chemerin-9 activates CMKLR1 with nanomolar activity (EC_50_ = 44 nM), [L^8^]-chemerin-9 is devoid of detectable activity at concentrations up to 10 µM.

### Chemerin-9 activates GPR1 in a loop conformation

**Fig. 6:**
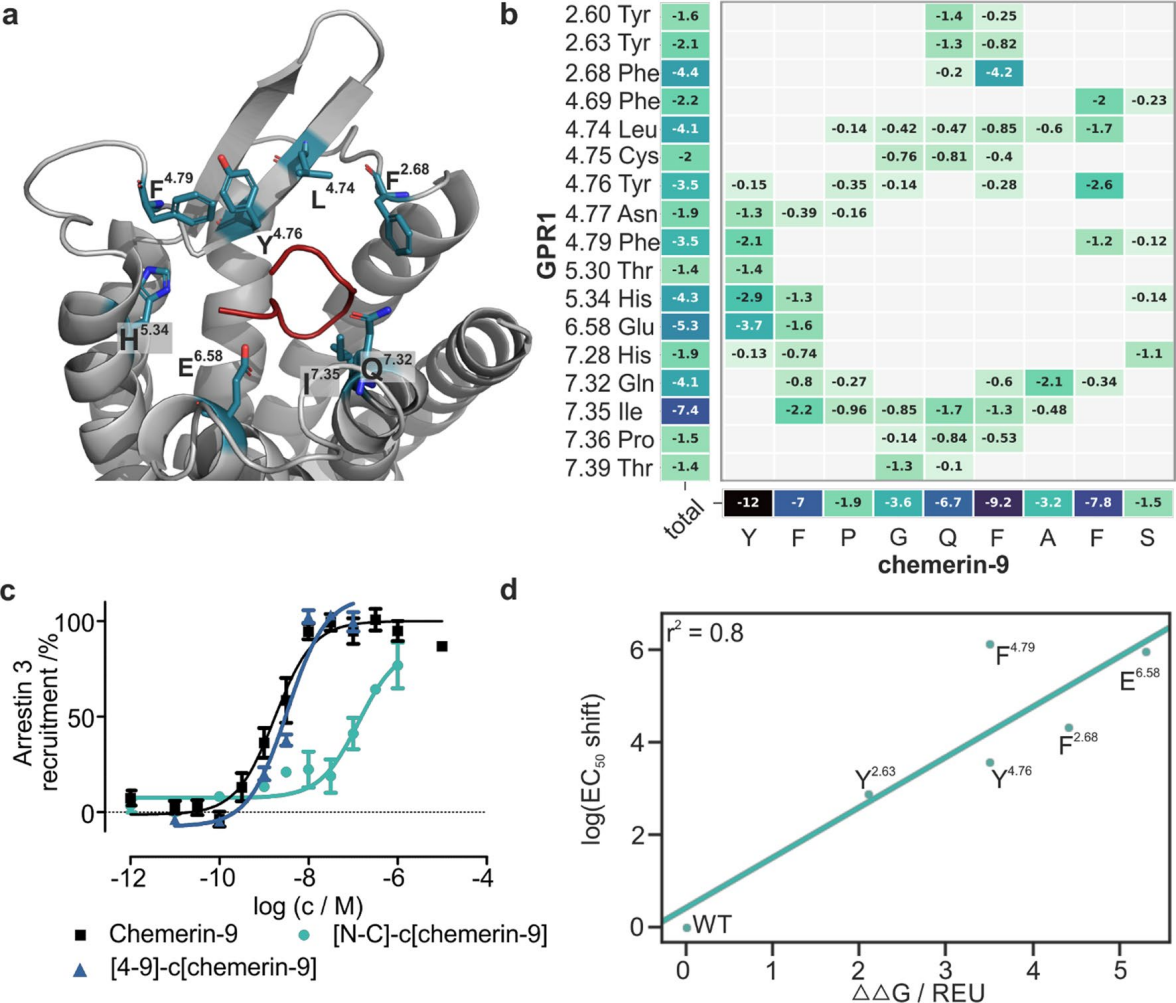
Binding mode of the Chemerin C-terminus at GPR1. **a** Best scoring model by interface score ΔG separated, with the ligand shown in red, the receptor in gray. Residues of the binding pocket are highlighted as sticks. **b** Contact map of the 20 best scoring modes by interface score ΔG separated, with darker colors indicating stronger interactions. Only receptor residues with <  – 1 REU are included. **c** Cyclized derivates [N–C]-c(chemerin-9) and [4-9]-c(chemerin-9) retain activity in an arrestin recruitment assay, confirming the loop-like conformation of the chemerin-9 terminus. **d** Predicted binding energies for several receptor residues strongly correlate with the logarithmic EC_50_ shifts of the respective alanine mutants, demonstrating that the models are in good agreement with the available experimental data

We suspected that chemerin-9 adopts a turn conformation for activating GPR1, which should allow for cyclization of the ligand while retaining activity. To test this hypothesis, we synthesized a highly constrained chemerin-9 derivate, connecting N- and C-terminus by a lactam bond ([N–C]-c(chemerin-9). This peptide displayed a significantly reduced potency but was still able to activate GPR1 (EC_50_ = 132 nM). To improve the potency at the receptor for potential therapeutic applications, we synthesized a second cyclic peptide with increased flexibility by exchanging positions 4 and 9 for D-homocysteine and cysteine. Oxidizing the peptide promoted the cyclization, forming a disulfide between positions 4 and 9 ([4-9]-c(chemerin-9)). This peptide showed high activity at GPR1 and was as active as the linear peptide (EC_50_ = 3.3 nM), as displayed in Fig. 6c.

### Restrained docking simulations uncover the binding mode of chemerin-9 at GPR1

With this data on the GPR1 ligand-binding mode at hand, we chose to construct a model of this interaction. Chemerin-9 was docked to the ten best scoring receptor models from each of the three largest clusters (i.e., a total of 30 models) using Rosetta FlexPepDock with our experimental data as restraints: all identified residues of the binding pocket (Fig. 3a) were required to interact with the ligand, residues V^4.67^ and F^4.69^ were restrained to interact with chemerin-9 residue F^8^ (Fig. 4). In addition, a loop conformation was enforced by a distance restraint between the ligand termini. The resulting models were clustered based on Cα RMSD, and the best scoring models from cluster 2 (Figure S3, selected based on their agreement with the experimental data) were subjected to Rosetta FastRelax without restraints. Finally, the 20 best scoring models by interface score ΔG separated were selected for analysis (Fig. 6b). In these models, chemerin-9 residue F^8^ interacts with a hydrophobic domain in the ECL2 consisting of F^4.69^, L^4.74^, Y^4.76^, and F^4.79^. Residue F^6^ displays a very pronounced interaction with F^2.68^ and, to a lesser extent, with Y^2.63^. Residue S^9^ is barely involved in binding to the receptor. The N-terminal residues Y^1^ and F^2^ interact with a wide range of residues in the receptor and highly contribute to the predicted binding energy. Chemerin-9 G^4^ and Q^5^ extend towards the bottom of the binding pocket and interact with T^7.39^. On the receptor side, I^7.35^ interacts with a range of ligand residues and displays the highest energy contribution of all receptor residues. Taken together, the interface between chemerin-9 and GPR1 is dominated by hydrophobic interactions. The predicted binding energies of different receptor residues show a high correlation with the logarithmic EC_50_ shifts of their respective alanine mutants (r^2^ = 0.8), demonstrating that the models are in good agreement with the available experimental data (Fig. 6d).

### GPR1 undergoes constitutive internalization

**Fig. 7:**
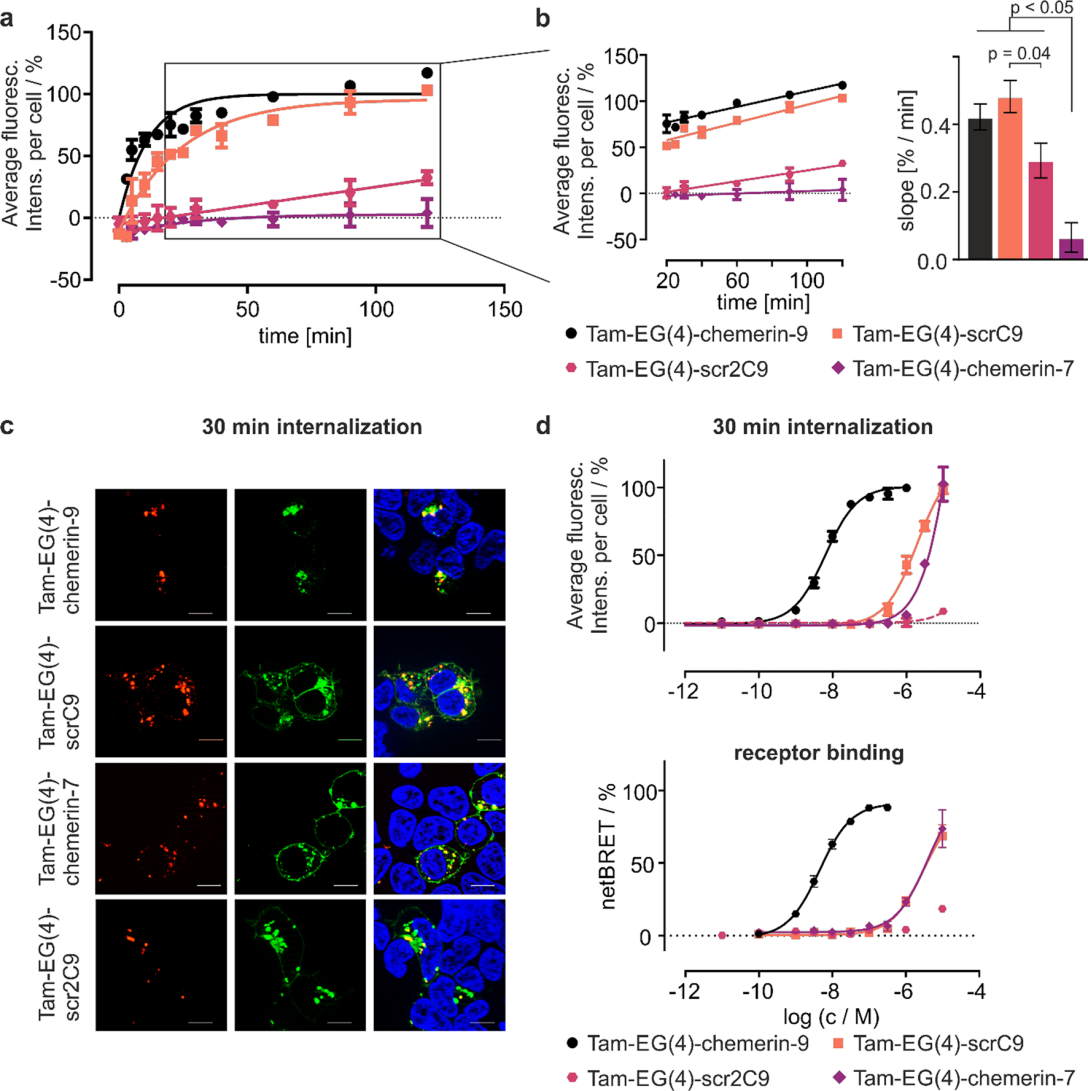
Various chemerin-derived peptides are internalized by GPR1.** a** HEK293 cells stably expressing GPR1 internalize Tam-EG(4)-chemerin-9 and the scrambled peptides Tam-EG(4)-scrC9 and Tam-EG(4)-scr2C9, but not Tam-EG(4)-chemerin-7. Cells were stimulated with 1 µM of the respective peptide; data points represent mean ± SEM from at least two independent experiments performed in duplicates. Intracellular accumulation of the labeled peptides over time was measured using a confocal microscopy high content imaging system. **b** After 20 min, the concentration of Tam-EG(4)-chemerin-9 and both scrambled peptides linearly increases over time. The slope of the linear regression for Tam-EG(4)-chemerin-7 does not significantly differ from zero, and is significantly smaller than the slope for Tam-EG(4)-chemerin-9, Tam-EG(4)-scrC9, and Tam-EG(4)-scr2C9 (p < 0.05, one-way ANOVA with Tukey’s multiple comparisons post-test). Bars represent mean ± SEM. **c** After stimulation with 1 μM peptide for 30 min, all internalized peptides co-localize with the GPR1-eYFP, demonstrating a receptor-dependent internalization. **d** Comparing concentration-dependent internalization in stably transfected HEK293-GPR1-eYFP cells and receptor binding to Nluc-GPR1-eYFP demonstrates that peptides need to bind to the receptor to be internalized

To characterize the function of GPR1 in the cell, HEK293 cells stably expressing GPR1-eYFP were treated with 1 µM of Tam-labeled peptides, and intracellular fluorescence was measured at distinct timepoints using an ImageExpress high content imaging system (Fig. 7). Stimulation with Tam-EG(4)-chemerin-9 led to a rapid, exponential accumulation of fluorescence in the cell within 15 min. This rapid accumulation was followed by a linear increase starting at around 20 min, which continues until the end of the experiment at 120 min. The truncated Tam-EG(4)-chemerin-7 peptide does not evoke any intracellular increase of fluorescence over time. In contrast, both scrambled peptides displayed substantial internalization. In the first 20 min, the growth of intracellular fluorescence for Tam-EG(4)-scrC9 is delayed compared to Tam-EG(4)-chemerin-9, but the linear increase between 20 and 120 min displays a comparable slope for both peptides (Fig. 7). A second scrambled peptide, Tam-EG(4)-scr2C9, did not display rapid intracellular accumulation in the first 15 min but showed a similar, linear increase of intracellular fluorescence as Tam-EG(4)-chemerin-9 and Tam-EG(4)-scrC9 (Fig. 7b). In contrast, neither of the scrambled peptides was internalized in CMKLR1-expressing HEK293 cells (Figure S4). Comparing the slopes of the linear regression lines of all peptides revealed a significant deviation from zero for Tam-(EG4)-chemerin-9, Tam-EG(4)-scrC9, and Tam-EG(4)-scr2C9, but not for Tam-EG(4)-chemerin-7 (*p* < 0.05). Moreover, the slope of the linear fit for Tam-EG(4)-chemerin-7 was significantly lower than for all other peptides.

To gain insights into the mechanisms of GPR1-mediated peptide internalization, we performed additional fluorescence microscopy experiments (Fig. 7c): after 30 min stimulation with 1 μM peptide, all peptides including Tam-EG(4)-chemerin-7 were found in intracellular vesicles co-localized with GPR1-eYFP. Thus, small amounts of Tam-EG(4)-chemerin-7 are internalized soon after stimulation, but this effect does not accumulate over time. Moreover, fluorescence microscopy revealed that stimulation with Tam-EG(4)-chemerin-9 and Tam-EG(4)-chemerin-7 induced internalization of the receptor. In contrast, GPR1-eYFP was still found in the membrane after stimulation with the two scrambled peptides Tam-EG(4)-scrC9 and Tam-EG(4)-scr2C9.

To further investigate the connection between binding and internalization of ligands, we performed concentration-dependent internalization and binding experiments (Fig. 7d). Regarding internalization, Tam-EG(4) displayed an EC_50_ of 6 nM, which is equal to the EC_50_ observed for this peptide in the ligand BRET-binding assay. Tam-EG(4)-scrC9 and Tam-EG(4)-chemerin-7 roughly display a 1000-fold increased EC_50_ in internalization and receptor binding, while 10 μM Tam-EG(4)-scr2C9 is needed to evoke a response in both assays.

**Fig. 8:**
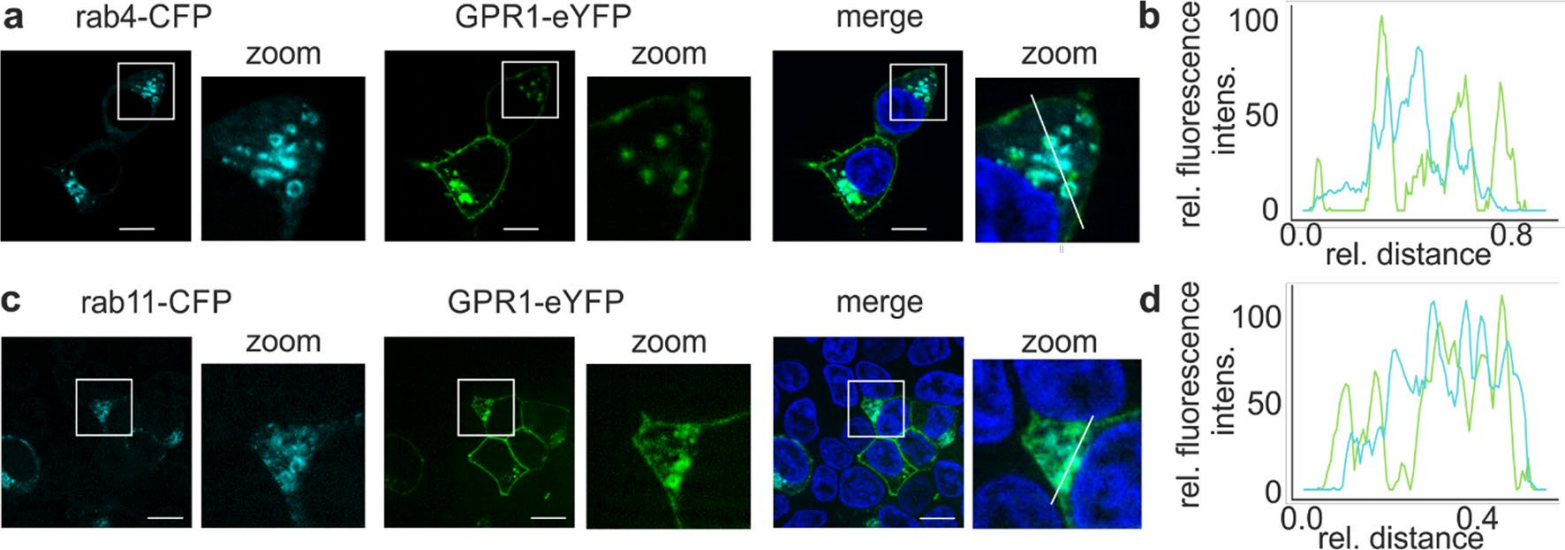
In the absence of ligand, GPR1 co-localizes with endosomal markers. **a** Live cell microscopy of HEK293 cells transiently transfected with GPR1-eYFP and rab4-CFP. **b** Co-localization of GPR1-eYFP (green) with rab4-CFP (cyan) was verified with a line scan. **c, d** Co-localization of GPR1-eYFP (green) with rab11-CFP (cyan) is even more pronounced. Shown is one representative cell from at least two independent experiments. Cell nuclei were stained with Hoechst 33342, scale bar = 10 µm

The scrambled scrC9 peptide is not able to induce arrestin recruitment to GPR1, but still accumulates in cells expressing GPR1. A fraction of GPR1 always resides in intracellular vesicles (Fig. 2b), which prompted us to investigate whether they co-localized with endosomal markers. We co-transfected GPR1-eYFP and rab4-CFP (marker for early endosomes) or rab11-CFP (marker for recycling endosomes) in HEK293 cells and examined this by live-cell fluorescence microscopy. Indeed, rab4-CFP forms distinct vesicular structures that co-localize with GPR1-eYFP (Fig. 8a). A one-dimensional intensity scan of CFP and eYFP fluorescence in imageJ demonstrated that the intracellular GPR1-eYFP was predominantly colocalized with rab4-CFP, and colocalization analysis using the R package colocR revealed a Pearson’s colocalization coefficient of 0.76 (Fig. 8b). Similarly, GPR1-eYFP colocalizes with rab11-CFP, confirmed by a line scan and a Pearson’s colocalization coefficient of 0.82 (Fig. 8c, d).

**Fig. 9:**
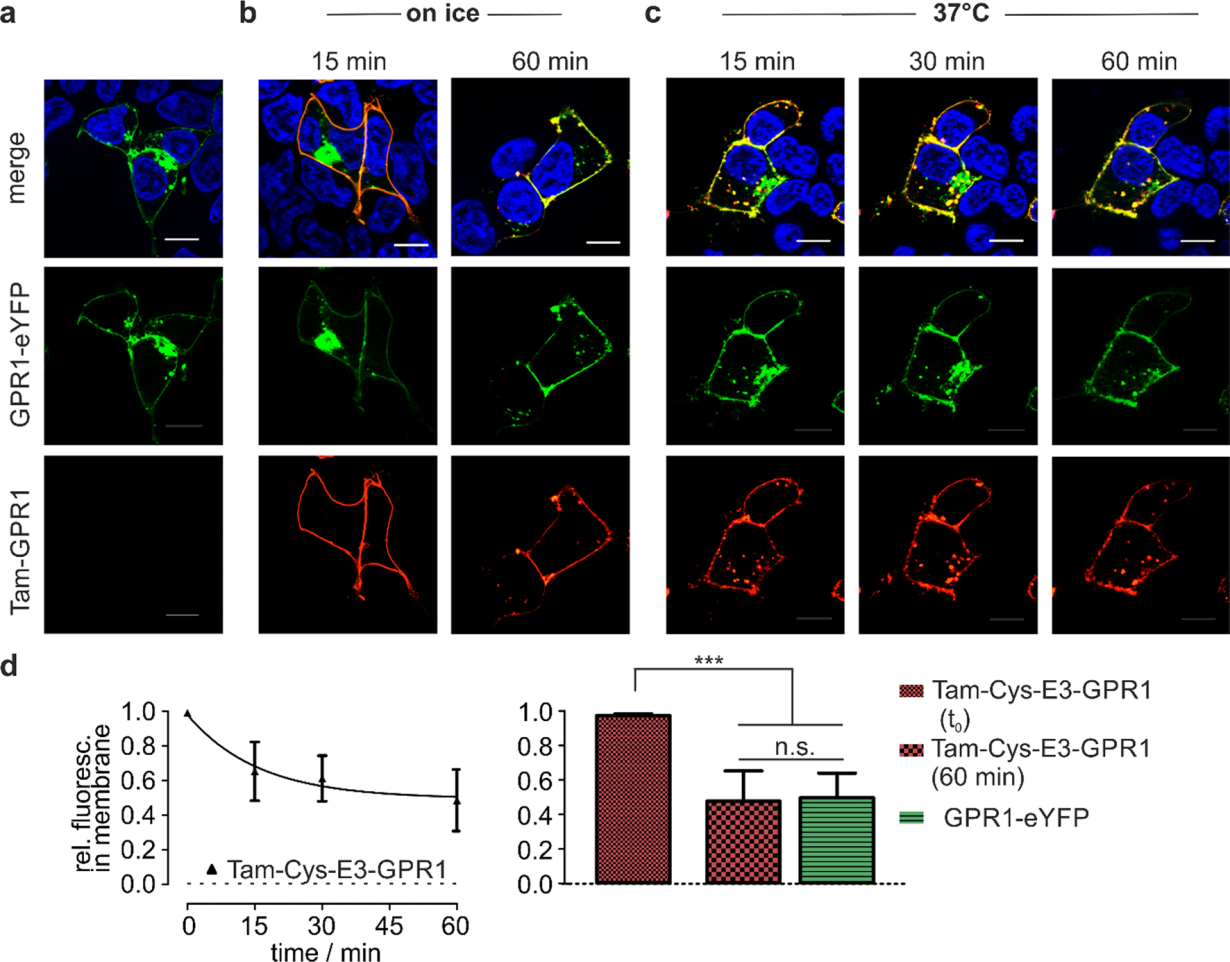
GPR1 shows rapid ligand-independent, constitutive internalization. **a** Cys-E3-tagged GPR1 is expressed in the membrane and intracellularly, similar to untagged GPR1. **b** After labeling and washing on ice to prevent internalization, only GPR1 in the membrane is Tam-labelled. **c** Already 15 min after the beginning of the labeling reaction, a significant amount of GPR1 has internalized. Images after 30 and 60 min show no further decrease of Tam fluorescence in the membrane, indicating that internalized receptors are recycled back to the membrane. Cell nuclei were stained with Hoechst 33342. Scale bar = 10 µm. **d** Quantification of rel. fluorescence intensity in the membrane and intracellular vesicles. After 60 min, 45% of Tam-fluorescence remains at the membrane, which matches the distribution of unlabeled GPR1-eYFP. Shown are mean ± SD of > 7 cells from at least two independent experiments. ***p < 0.0001 in a one-way ANOVA with Tukey’s post-test

We hypothesized that GPR1 undergoes constitutive internalization and recycling, independent of the presence of ligand. To test this hypothesis, we selectively labeled cell surface receptors by peptide-templated transfer of a fluorescent dye [30, 31]. We genetically introduced an N-terminal Cys-E3 tag into GPR1, which specifically interacts with a synthetic peptide probe (termed K3) by coiled-coil interactions. This high-affinity interaction brings the free N-terminal cysteine of the Cys-E3 tag in proximity to a Tam fluorophore, which is bound to the K3 peptide probe by a thioester. A transthioesterification followed by an S–N acyl shift transfer the Tam dye to the N-terminus, forming a stable amide bond between receptor N-terminus and dye [31]. This approach allows to discriminate receptors embedded in the cell membrane from intracellular subpopulations at the time of reaction. Comparing eYFP fluorescence revealed that unlabeled, Cys-E3-tagged GPR1-eYFP resides in intracellular vesicles and the cell membrane, similar to untagged GPR1-eYFP (Fig. 9a). To prevent receptor endocytosis, we performed the peptide-templated labeling and subsequent washing steps on ice, followed by microscopy with the cooled down sample carrier. Images of cells on ice were taken up to 15 min post-labeling and summarized as timepoint t_0_ (Fig. 9d).This visualizes only receptors in the membrane (Fig. 9b, lower panel). When the labeling reaction was performed at 37 °C, significant amounts of labeled receptor had already internalized 15 min after starting the reaction (Fig. 9c). We quantified the relative distribution of Tam fluorescence in the membrane and intracellular vesicles over time (Fig. 9d): after 15 min, 65 ± 18% (mean ± SD) of Tam fluorescence is still in the membrane, and after 30 min, 60 ± 14% are left. The distribution of labeled GPR1 reaches an equilibrium at around 60 min when 45 ± 16% of Tam fluorescence is in the membrane. This distribution of Tam fluorescence after 60 min matches the distribution of eYFP fluorescence for untagged GPR1-eYFP (50 ± 14%). In contrast, after 60 min on ice, the large majority of Tam-labeled GPR1 was still localized in the membrane (Fig. 9b).

## Discussion

Chemerin is a signaling protein involved in several physiological processes, including adipogenesis, host defense, and reproductive functions [13]. An imbalance in the regulation of these processes induces severe effects, and consequently, chemerin itself underlies tight control. This control is most striking on the level of proteolytic activation and inactivation, but also on the receptor level. Three chemerin receptors GPR1, CMKLR1, and CCRL2 display distinct expression patterns and pharmacology, indicating a complex interplay between these receptors and their shared ligand.

GPR1 lacks functional coupling to G proteins but rapidly recruits arrestin3 in response to stimulation with chemerin. The C-terminus of chemerin is responsible for activating the receptor, as described for CMKLR1. Our BRET ratios of GPR1 correspond to the values obtained by de Henau et al*.* in a similar setup [18]. Interestingly, GPR1 recruits arrestin3 to the membrane upon stimulation with ligand, but this interaction seems to be transient: while arrestin3 stays at the membrane, GPR1 internalizes into intracellular vesicles. Receptors that dissociate from arrestin at the membrane before internalization are classified as class A, which, in general, rapidly recycle back to the membrane after activation [32, 33]. Next, we examined the ability of truncated chemerin proteins and peptides to activate GPR1. ChemS157 and ChemF156 display the same activity, indicating that these two proteins have a same activity profile for CMKLR1 and GPR1. The C-terminal part of chemerin is conserved across 9 mammalian species and is crucial for binding to CMKLR1 [34]. Thus, we expected the same region to contribute to GPR1 activation as well. Peptides derived from the respective C-termini of ChemS157 and ChemF156 show no decreased activity, chemerin-9 (chemerin^149−157^) displays the same potency as the corresponding full-length protein (Table 1). The minimal activation sequence starts at Y^149^, removal of this residue and the following F^150^ leads to a significant loss of activity, similar to results obtained at CMKLR [35]. In line with previous results, this demonstrates that the C-terminus of chemerin is the dominant part of the protein that binds to GPR1 [2, 18]. A peptide lacking the C-terminal S^157^ still showed no loss of activity, but further truncation of the sequence by removing F^156^ almost completely abolished activity. Chemerin^149−155^ (chemerin-7) induced only marginal arrestin recruitment starting at 1 µM. The same residue is critical for the activation of CMKLR1 [35]. Hence, chemerin species lacking the last phenylalanine display no activity at either GPR1 or CMKLR1. Total levels of chemerin increase with the body-mass index, and the ratio of the different chemerin forms (prochemerin, ChemS157, ChemF156, and ChemA155) differ between lean and obese subjects [36, 37]. Interestingly, a previous study performed in mice demonstrated a higher bioactivity ratio (active chemerin/total chemerin) in mice fed a high-fat diet when determined by GPR1 activation, but not measured by CMKLR1 activation [38].

The chemerin C-terminus is responsible for the activation of GPR1 and CMKLR1. Based on the high sequence homology of these two receptors, we hypothesized that they might share a common binding mode. Based on our previous results on the binding mode of chemerin-9 at CMKLR1, we selected several residues in the ECLs of GPR1 for investigation. We exchanged Y^2.63^, F^2.68^, F^4.76^, F^4.79^, and E^6.58^ for alanine and examined the influence of these mutations in a nanoBRET-based binding assay (Fig. 3). As expected, all mutations had a significant impact on ligand binding, confirming that chemerin-9 occupies approximately the same sites in GPR1 and CMKLR1. An essential interaction in the binding mode of chemerin-9 at CMKLR1 is between the ultimate phenylalanine in the ligand and a hydrophobic pocket formed by the ECL2 of the receptor. To investigate whether this interaction occurs in GPR1 as well, we performed 2D mutagenesis experiments. In CMKLR1, positions 4.67 and 4.69 consist of alanine and leucine, respectively. Exchanging these for a valine and a phenylalanine decreased the EC_50_ shift between chemerin-9 and [L^8^]-chemerin-9 by tightening the hydrophobic pocket formed by ECL2 [27]. Now, we reversed this experiment by introducing the V^4.67^A_F^4.69^L mutants into GPR1. Indeed, while [L^8^]-chemerin-9 is as active as chemerin-9 at the wild-type GPR1, a small shift at the V^4.6^7A_F^4.69^L mutant (Fig. 4) can be found. Thus, the ECL2 represents the binding site for F^8^ in both receptors. The fact that the ECL2 forms a tighter hydrophobic pocket in GPR1 than in CMKLR1 can be exploited for the rational design of selective ligands: as a proof of concept, [L^8^]-chemerin-9 displays a high potency at GPR1 (EC_50_ = 1.4 nM), but does not induce arrestin recruitment at CMKLR1 (Fig. 5). It is important to note, however, that [L^8^]-chemerin-9 does induce G protein-signaling at CMKLR1, although with a 20-fold loss of activity compared to chemerin-9 [27]. Conversely, ligands with larger side chains in position 8 may be suitable for the design of selective ligands targeting CMKLR1.

We previously showed that cyclic chemerin-9 derivatives activate CMKLR1 with high potency, and the same holds true for GPR1 (Fig. 6) [27]. This highlights that the binding mode of the chemerin C-terminus is highly related at both receptors. It also demonstrates that cyclization is a suitable approach to stabilize rationally designed ligands for GPR1.

Activation of GPR1 by chemerin-9 requires a specific conformation of the ligand. Surprisingly, however, two Tam-labeled scrambled peptides that are both unable to activate GPR1 are still internalized by GPR1 (Fig. 7). This discrepancy between ligand-induced arrestin recruitment on the one hand, and efficient receptor-mediated uptake on the other, is a strong indication for an activation-independent internalization mechanism [39]. In addition, the fact that the intracellular concentration of the wild type and both scrambled peptides continues to increase during extended periods indicates that receptors recycle back to the membrane after internalization. This hypothesis is supported by our findings that GPR1 co-localizes with rab4 and rab11, even in the absence of ligand (Fig. 8), as rab4 and rab11 are characteristic for the early and recycling endosome, respectively [40]. Ultimately, we used an approach to specifically label cell surface receptors by a peptide-templated acyl transfer [31]. This technique allowed to follow a specific receptor population with high spatiotemporal resolution [30]. We demonstrate by this approach as well that receptors spontaneously internalize without ligand stimulation (Fig. 9). The fact that the ratio of internalized to membrane-bound receptors quickly reaches an equilibrium further supports the idea that internalized GPR1 rapidly recycles back to the membrane. These results demonstrate that any molecule that binds to GPR1 is scavenged and brought into the cell by the receptor, regardless of whether it induces GPR1 activation. The receptor then quickly recycles back to the membrane, making it perfectly adapted to decrease extracellular concentrations of chemerin. Importantly this constitutive internalization is not accompanied by constitutive activity [41].

The ligand-binding modes are highly conserved between CMKLR1 and GPR1. Therefore, any agonist or antagonist of CMKLR1 is likely to be bound and internalized by GPR1. There are few examples of cell types that express both GPR1 and CMKLR1: macrophages generally express CMKLR1, but resident alveolar macrophages additionally display GPR1 mRNA expression [42]. Interestingly, chemerin is described to mediate anti-inflammatory functions in a murine lung model of lung disease [24]. Chemerin plays an essential role in adipose tissue, and CMKLR1 expression was previously demonstrated in adipocytes [19]. GPR1 expression was found in the stromal vascular fraction of adipose tissue, and hence adipose tissue macrophages may be another population of immune cells that have GPR1 and CMKLR1 at their disposal [43].

Our results that both scrambled chemerin-9 peptides are internalized by GPR1 to varying degrees may indicate that this receptor scavenges not only chemerin but also related peptides or proteins. Atypical chemokine receptors show many similarities to GPR1. They are essential for the regulation of chemokine activity and lack classical G protein signaling [44]. Meyrath et al*.* previously published a study demonstrating that the atypical chemokine receptor 3 (ACKR3, formerly CXCR7) not only binds chemokines, but also a broad range of opioid peptides [45]. Considering that GPR1 is expressed in the brain, a similar role for this receptor is possible [1]. Indeed, a previously published study states that the neuropeptide FAM19A1 may be an additional ligand for GPR1 [46].

The closest homologs of the chemerin receptors GPR1 and CMKLR1 are the receptors for the chemotactic protein complement 5 a, namely, C5a receptor 1 (C5aR1) and C5a receptor 2 (C5aR2) [47]. They show similar characteristics, with C5aR1 being a classical, G_i_-coupled receptor that mediates chemotaxis of immune cells, and C5aR2 being a constitutively internalizing scavenging receptor that does not signal through G proteins [48]. In contrast to GPR1, however, C5aR2 does not co-localize with rab4 and seems to recycle at a slower rate [49]. GPR1, C5aR2, and the atypical chemokine receptors all display mutated DRY motifs in TM3. However, a study on the C5aR2 showed that mutation of this motif is not responsible for the lack of G protein coupling and a similar study on ACKR2 (formerly known as D6) found the same [50, 51]. Not surprisingly, mutation of the altered DRY motif (DHY) in GPR1 did not restore G protein signaling [43]. The molecular basis for the lack of G protein signaling, therefore, remains unclear.

Taken together, our results demonstrate that the C-terminus of chemerin displays a common binding mode at the two receptors GPR1 and CMKLR1. Differences in the ECL2 of these receptors have been exploited to design the first GPR1-selective ligand and may be useful for the rational design of CMKLR1-selective ligands as well. A cycle of ligand-independent internalization followed by rapid recycling makes GPR1 perfectly adapted as a scavenging receptor, and our results indicate that this role of GPR1 may not be restricted to chemerin.

## Materials and methods

### Materials

#### Peptide synthesis

Fmoc-protected amino acids were purchased from ORPEGEN (Heidelberg, Germany). Fmoc-D-homocysteine(Trt)-OH and Fmoc-NH-PEG(4)-OH were purchased from Iris Biotech (Marktredwitz, Germany). Peptide resins, 1-hydroxy benzotriazole (HOBt), ethanedithiol (EDT), diethyl ether, and trifluoracetic acid (TFA) were obtained from Merck (Darmstadt, Germany). N,N’-diisopropyl carbodiimide (DIC) and 2-cyano-2-(hydroxyimino) acetic acid ethyl ester (oxyma) were purchased from Iris Biotech (Marktredwitz, Germany). Dimethylformamide (DMF) and dichloromethane (DCM) were purchased from Biosolve (Valkenswaard, Netherlands), acetonitrile (ACN) was obtained from VWR (Darmstadt, Germany). O-(7-Azabenzotriazol-1-yl)-N,N,N,N-tetramethyluronium hexafluorophosphate (HATU), N,N-diisopropylethylamine (DIPEA), piperidine, and thioanisole (TA) were obtained from Sigma-Aldrich (St. Louis, USA). 6-Carboxytetramethylrhodamine (Tam) was purchased from emp biotech (Berlin, Germany).

#### Cell culture

Cell culture media (Dulbecco’s Modified Eagle’s Medium (DMEM), Ham’s F12), as well as trypsin–EDTA, Dulbecco’s Phosphate-Buffered Saline (DPBS), and Hank’s Balanced Salt Solution (HBSS), were obtained from Lonza (Basel, Switzerland). Fetal bovine serum (FBS) was from Biochrom GmbH (Berlin, Germany). Hygromycin B was purchased from Invivogen (Toulouse, France), and Opti-MEM was obtained from Life Technologies (Basel, Switzerland). LipofectamineTM 2000 was obtained from Invitrogen (Carlsbad, CA, USA). MetafecteneProTM was received from Biontex Laboratories GmbH (München, Germany). Coelenterazine H was purchased from DiscoverX (Fremont, CA, USA), Hoechst33342 nuclear stain was obtained from Sigma-Aldrich (St. Louis, MO, USA). Bovine arrestin-3 was fused to Rluc8 and cloned into pcDNA3 vector for BRET studies. Primers for PCR were bought from Biomers (Ulm, Germany). Furimazine was purchased from Promega (Madison, WI, USA).

### Peptide synthesis

All peptides were synthesized by 9-fluorenylmethoxycarbonyl/tert-butyl (Fmoc/^t^Bu) solid-phase peptide synthesis strategy on a scale of 15 µmol on a Wang resin preloaded with the first amino acid, or on a 2-chlorotrityl chloride resin. All reactions were performed at rt unless stated otherwise. All standard amino acids were coupled using a Syro II peptide synthesizer (MultiSynTech, Bochum, Germany). Coupling reactions were performed twice with 8 equiv of the respective, Fmoc-protected amino acid activated in situ with equimolar amounts of oxyma and DIC in DMF for 30 min. Fmoc-removal was achieved by reaction with 40% (v/v) piperidine in DMF for 3 min and 20% (v/v) piperidine in DMF for 10 min, the resin was washed with DMF and DCM after every reaction. Fmoc-D-hCys(Trt)-OH and Fmoc-PEG(4)-OH were manually coupled to the peptide by reaction with 5 equiv and equimolar amounts of HOBt and DIC in DMF overnight. N-terminal Tam-labeling was achieved by reaction with 2 equiv Tam, 1.9 equiv HATU, 2 equiv DIPEA in DMF for 2 h.

[N–C]-c(chemerin-9) was synthesized on a 2-chlorotrityl chloride resin, the first amino acid was coupled to the resin by reaction with 1.5 equiv Fmoc-Ser(tBu)-OH, 6 equiv DIPEA in DCM overnight. All following amino acids were coupled as described above. After protected cleavage from the resin with 10% (v/v) acetic acid, 10% (v/v) trifluoroethanol in DCM for 2 h at rt, a lactam bond between the N- and C-terminus was formed by incubation with 5 equiv HOBt, DIC in DCM for 72 h at rt.

All peptides were cleaved by reaction with TFA/EDT/TA (90:3:7, v:v:v) for 3 h and precipitated in ice-cold diethyl ether/hexane (1:3). After full cleavage, the crude peptides were precipitated from cold diethyl ether/hexane at  – 20 °C for at least 3 h, washed with diethyl ether and collected by centrifugation. The thiol-containing peptide was dissolved in refolding buffer (20% ACN, 0.15 M NaCl, 25 mM Tris, pH 7.7) and incubated at rt for 72 h for cyclization to yield the disulfide containing peptide [4–9]-c(chemerin-9). All peptides were purified by RP-HPLC on a Kinetex 5 µm XB-C18 100 Å column (Phenomenex, Torrence, USA), purity and identity were confirmed by RP-HPLC on a Jupiter 4 µm Proteo 90 Å C12 (Phenomenex), MALDI-ToF MS on an Ultraflex II and ESI MS on an HCT ESI (Bruker Daltonics, Billerica, USA). RP-HPLC was performed employing linear gradients of eluent B (0.08% TFA in ACN) in eluent A (0.1% TFA in H_2_O).

### Protein expression

Full-length chemerinS157 was produced as a His_10_-fusion protein in *E.coli* BL21 (DE3) as described previously [52]. In brief, the plasmid DNA (His_10_-chemerinS157 in pET16b) was transformed into *E.coli*. Bacteria were grown in LB medium supplemented with 0.1 mg/mL ampicillin. Upon reaching OD_600_ = 0.8, expression was induced with 1 mM isopropyl β-D-thiogalactopyranoside (IPTG). After 6 h expression at 37° C, cells were harvested by centrifugation. Resuspended in base buffer (0.5 M NaCl, 25 mM Tris/HCl, pH 7.8), the cells were lysed using a FastPrep-24 bead beating lysis system (MP Biomedicals, Irvine, USA). After washing with base buffer supplemented with 2 M urea, inclusion bodies were solubilized in base buffer containing 8 M urea. The solubilized protein was purified by immobilized metal affinity chromatography and refolded by stepwise dialysis employing decreasing urea concentrations and a cysteine/cystamine redox pair. Purity and identity were confirmed by SDS-PAGE, RP-HPLC, and MALDI ToF MS (Ultraflex III, Bruker).

### Mutagenesis

All mutations were introduced into an hGPR1-eYFP pVitro2 plasmid kindly provided by Stefan Schultz [52]. All PCR reactions were carried out using Phusion polymerase. The SigP-Cys-E3-hGPR1-eYGP construct was cloned by PCR utilizing the primers shown in Table 2 by introducing the Cys-E3 tag and the signal peptide in two sequential PCR reactions. The Nluc-GPR1-eYFP construct was cloned by overlap extension PCR using the primers display in Table 2, exploiting the *MluI* and *XbaI* restriction sites. The secNluc-pNL1.3 vector was purchased from Promega. Point mutations were introduced into the Nluc-GPR1-eYFP construct using the QuickChange mutagenesis protocol with primers carrying the respective mutations. The success of any mutagenesis or cloning reactions was verified by Sanger sequencing.

**Table 2:** Primers used in PCR reactions to introduce either the E3 tag and the signal peptide for increased membrane expression, or the N-terminal Nluc for BRET-based binding assays

Primer	Sequence
E3-tag forward	**ATGTGCGAGATCGCCGCCCTGGAGAAGGAGATCGCCGCCCTGGAGAAGGAGATCGCCGCCCTGGAGAAGGGCGGCTCA**ATGGAAGATTTGGAGGAAACATTATTTG
E3-tag reverse	**GCGATCTCGCACAT**GGTGGCACGCGTGG
SigP forward	**ATGCAGCCGCCTCCAAGTCTGTGCGGACGCGCCCTGGTTGCGCTGGTTCTTGCCTGCGGCCTGTCGCGGATCTGGGGATGC**GAGATCGCCGCCCTGGAG
SigP reverse	**GAGGCGGCTGCAT**GGTGGCACGCGTGG
Nluc-forward	ATATACGCGTGCCACCATG**AACTCCTTCTCCACAAGCGCC**
Nluc-linker-reverse	**GCTGCCTCCGCCTCCGCTCGCCAGAATGC**
linker-GPR1-forward	**GGAGGCGGAGGCAGC**GAAGATTTGGAGGAAACATTATTTGAAG
GPR1-eYFP-reverse	**ATATTCTAGACTA**CTTGTACAGCTCGTCCATGCCGAG

Parts in bold correspond to the sequences not present in the respective template

### Cell culture

HEK293 and COS-7 cells were cultivated in DMEM/Ham’s F12 supplemented with 15% FBS or DMEM supplemented with 10% FBS, respectively. HEK293 and COS-7 cells were authenticated by DNA barcoding in 2017. HEK293 cells stably transfected with GPR1-eYFP were cultivated in DMEM/Ham’s F12, 15% FBS supplemented with 100 µg/mL hygromycin. All cells were cultivated in T75 cell culture flasks at 37 °C, 95% humidity, 5% CO_2_. All experiments with eukaryotic cells were carried out at 37 °C unless stated otherwise.

### Characterization of arrestin recruitment

Arrestin recruitment was characterized by measuring the bioluminescence resonance energy transfer (BRET) ratio between luciferase-tagged arrestin3 and either GPR1-eYFP or CMKLR1-eYFP as described previously [53]. In brief, COS-7 cells in 75 cm^2^ cell culture flasks were transiently transfected overnight with 7800 ng GPR1-eYFP or CMKLR1-eYFP in pVitro2 and 200 ng Rluc8-Arr3 in pcDNA3 using MetafectenePro according to the manufacturer’s protocol. One day post-transfection, cells were detached using trypsin/EDTA, resuspended in 20 mL DMEM/Ham’s F12 w/o phenol red supplemented with 15% FBS and seeded in white 96 well plates (100 µL cell suspension/well) and grown overnight. Prior to the assay, the medium was replaced by 100 µL BRET buffer (25 mM HEPES, pH 7.4 in HBSS), and 50 µL of the *Renilla* luciferase substrate Coelenterazine H (Nanolights) was added (final concentration 4.2 µM), followed by 5 min incubation. For kinetic measurements, cells were stimulated with agonist in buffer or buffer alone, and fluorescence was measured for 20 min using a Tecan Infinite M 200 Reader (Tecan, Männerdorf, Switzerland) using the filter sets Green1 (YFP emission, luminescence 520–570 nm) and Blue1 (luciferase luminescence 370–480 nm). For concentration–response curves, cells were stimulated with agonist or blank, and luminescence was measured after 10 min. BRET signal was calculated as the ratio of fluorescence divided by luminescence, netBRET signal was calculated by subtracting the BRET signal of unstimulated wells from the respective samples.

### Ligand BRET

Ligand binding was characterized using a NanoBRET approach with Tam-labeled ligands and hGPR1-eYFP N-terminally modified with a NanoLuc [54]. COS-7 cells in 25 cm^2^ cell culture flasks were transfected with 4000 ng of the respective Nluc-GPR1-eYFP construct using MetafectenePro according to the manufacturer’s protocol. One day post-transfection, cells were detached using trypsin/EDTA, resuspended in 10 mL phenol red-free DMEM/Ham’s F12, 15% FBS, seeded into solid-black 96 well plates (100 µL/well), and grown overnight. Before the assay, the medium was replaced by 100 µL BRET buffer. Cells were stimulated with Tam-labeled peptides, and 50 µL of furimazine in BRET buffer was added. Measurements were performed using a Tecan Spark plate reader (Tecan, Männerdorf, Switzerland), measuring Tam-emission (550–700 nm), and NanoLuc luminescence (430–470 nm). BRET and netBRET signals were calculated as described above.

### Live-cell fluorescence microscopy

For fluorescence microscopy, HEK293 cells were seeded into 8 well 15µ-slides (Ibidi, 140,000 cells/200 µL/well) coated with poly D-lysine and grown overnight. Next, cells were transfected with 900 ng of the respective GPR1-eYFP plasmid and, where applicable, 100 ng of either rab4-CFP, rab11-CFP, or mCherry-arrestin3. Transfection was achieved using Lipofectamine 2000 according to the manufacturer’s protocol. One day post-transfection, fluorescence microscopy experiments were performed on an AxioVision Observer.Z1 microscope equipped with an ApoTome imaging system (Zeiss, Jena, Germany). Before the experiment, cells were starved in OptiMEM reduced serum medium containing Hoechst 33342 for 30 min. To observe arrestin recruitment, cells were stimulated with 1 µM chemerin-9 in OptiMEM for the indicated period. To observe peptide uptake, cells were stimulated with 1 µM Tam-chemerin-9 in OptiMEM, which was replaced with acidic wash (50 mM glycine, 100 mM NaCl, pH 3) in HBSS after the indicated time, followed by two washing steps with OptiMEM. Microscopy was carried out in OptiMEM; the exposure time was held constant whenever changes over time were observed.

### Peptide-templated on-surface labeling

Peptide-templated acyl transfer for selective labeling of membrane receptors was carried out as described previously [30]. Because E3-tagged GPR1 was not expressed in the membrane, the endothelin B receptor N-terminal signal peptide was attached. This signal peptide improves transport to the membrane and is cleaved upon successful membrane integration [55]. HEK293 cells were seeded out and transfected with SigP-Cys-E3-GPR1-eYFP in pVitro2 as described above and incubated in Hoechst 33342 in OptiMEM for 30 min, followed by 10 min incubation in 20 mM HEPES in HBSS, pH 7 (labeling buffer). Labeling of cell surface receptors was achieved by 5 min incubation with 150 nM Tam-K3 peptide probe in labeling buffer supplemented with 0.1 mM TCEP. The cells were washed by incubation with 200 mM NaHCO_3_ in DPBS w/o Ca^2+^ and Mg^2+^, pH 8.5 for 1.5 min, followed by two washing steps with labeling buffer. Microscopy studies were finally carried out in labeling buffer as described above. To prevent receptor internalization, the cells were cooled on ice, followed by labeling and washing with ice-cold solutions and microscopy at rt. The Tam-K3 peptide was synthesized as described before [31]. The fraction of Tam-fluorescence in the membrane was determined by quantifying total and intracellular fluorescence for each cell individually in imageJ. The fluorescence fraction in the membrane was defined as the difference between intracellular and total fluorescence divided by total fluorescence.

### Peptide uptake using a high content imaging system

To quantify receptor-mediated peptide uptake, the intracellular accumulation of Tam-fluorescence was observed at different timepoints. HEK293 cells stably transfected with GPR1-eYFP were seeded into µclear, black 96 well plates (100,000 cells/well) coated with poly D-lysine and grown overnight. Before the experiment, cells were incubated with Hoechst 33342 in OptiMEM, which was replaced with pure OptiMEM after 30 min. Cells were stimulated with 1 or 10 µM of the respective, Tam-labeled peptide for the specified periods, followed by washing with acidic wash (50 mM glycine, 100 mM NaCl, pH 3) in HBSS. Microscopy was carried out in OptiMEM using an ImageXpress Micro Confocal High-Content Imaging System (Molecular Devices, San José, United States), using the appropriate filters for the respective fluorophores. The fluorescence intensity per cell was automatically analyzed for each well by a module detecting the nuclei (5–30 µm in diameter and 100 Gy levels above background) and the granules by Tam-peptide fluorescence (2–5 µm in diameter, 70 Gy levels above background).

### Statistical analysis

Linear and nonlinear regression, statistical analysis, and calculation of mean, SEM or SD was carried out using GraphPad Prism 8 except for BRET kinetics, where mean ± 95% CI values were plotted using seaborn and matplotlib in python 3. Linear regression of ΔΔG vs log(EC_50_) values was calculated in python 3 using the scipy stats module. The applied statistical tests, including sample sizes, are given in the respective figure legends.

### Homology modeling

Homology models of GPR1 in the *apo* state were produced using RosettaCM [56] with the crystal structures of C5aR1 [57] (PDB: 6c1r), CCR9 [58] (PDB: 5lwe), CXCR4 [59] (PDB: 3odu), APJR [60] (PDB: 5vbl) and AT1R [61] (PDB: 4zud) as templates as described previously [27, 28]. In brief, crystal structures were stripped of fusion proteins, ions, etc., followed by threading of the CMKLR1 sequence onto the template structures according to the sequence alignment given in Table S1. The threaded templates were hybridized giving a chimeric template, which was subjected to a Monte Carlo-based energy minimazion. Rosetta 3.9 was used for all modeling steps, 1500 homology models were produced in total.

### Peptide docking

To include structurally diverse templates for peptide docking, the homology models were clustered based on C_α_ RMSD, and chemerin-9 was docked into the ten best scoring models by total score from each of the three largest clusters using Rosetta FlexPepDock ab initio [62]*.* One sided restraints for the identified binding residues were applied to keep the peptide in the binding pocket, and a loop conformation of the peptide was enforced by a distance restraint between the peptide N- and C-terminus. An interaction between chemerin-9 residue F^8^ and CMKLR1 residues V^4.67^ and F^4.69^ was enforced by a distance restraint. In total, 25,000 models were produced. After clustering, the best scoring models from the cluster that best represented the experimental data were energy minimized using Rosetta FastRelax. The 20 best scoring final models by interface score ΔG separate were analyzed using a per residue energy breakdown. A detailed description of the modeling and docking process is given in the Supplementary Information.

### Supplementary Information

Below is the link to the electronic supplementary material.

Supplementary file1 (PDB 395 KB)

Supplementary file2 (PDF 482 KB)

## Data Availability

Models of the docked GPR1–chemerin-9 complexes are available as part of the supplementary material.

## Code availability

All code used for homology modeling and peptide docking is available as part of the supplementary information.
