# Supplementary material for: Ligand-binding and -scavenging of the chemerin receptor GPR1
Source: Cell Mol Life Sci. 2021 Jul 9;78(17-18):6265–81. doi: 10.1007/s00018-021-03894-8 (PMC8429170; doi:10.1007/s00018-021-03894-8)
Supplement: Supplementary file 2 — Supplementary file2 (PDF 482 KB) [file 18_2021_3894_MOESM2_ESM.pdf]

Supplementary Information

Ligand-Binding and -Scavenging of the Chemerin Receptor GPR1

Tobias F. Fischer, Anne S. Czerniak, Tina Weiß, Clara T. Schoeder, Philipp Wolf, Oliver Seitz, Jens Meiler, Annette G. Beck-Sickinger

Supplementary Results

Arrestin Recruitment

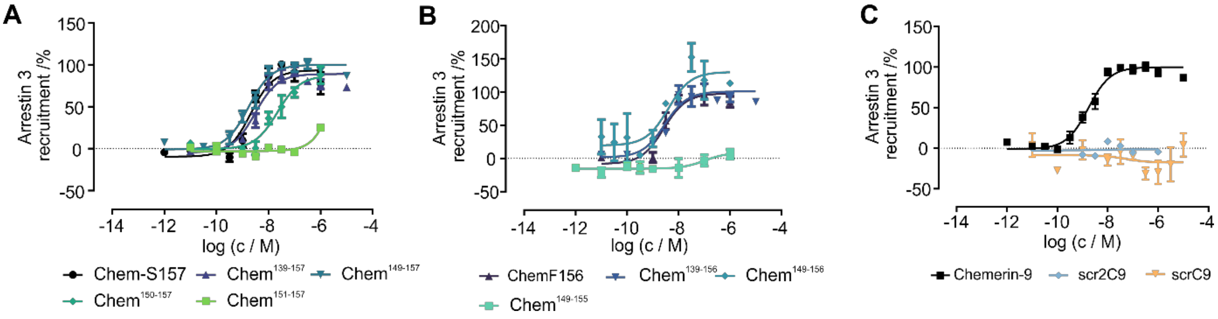

Figure S1: *BRET profiles for chemerin and derived peptides. Data points represent mean ± SEM from at least two independent experiments performed in quadruplicates.* ***A)*** *N-terminal truncations beyond Y^{149} result in a loss of activity.* ***B)*** *C-terminal truncations beyond F^{156} result in a completely inactive peptide.* ***C)*** *The scrambled peptides scrC9 and scr2C9 do not induce arrestin recruitment to GPR1.*

Molecular Modeling

In a first step, 1500 homology models of GPR1 based on the crystal structures of CCR9 (5lwe), APJR (5vbl), C5aR1 (6c1r), CXCR4 (3odu), and AT1R (4zud) were produced. Table S1 displays the alignment of the receptors used for producing the homology models. The models formed three major and three minor clusters (Figure S2), which differed mainly in the extracellular loops 2 and 3. To include structural heterogenicity, we used the 10 best scoring models from clusters 1, 2, and 3 as templates for docking the ligand.

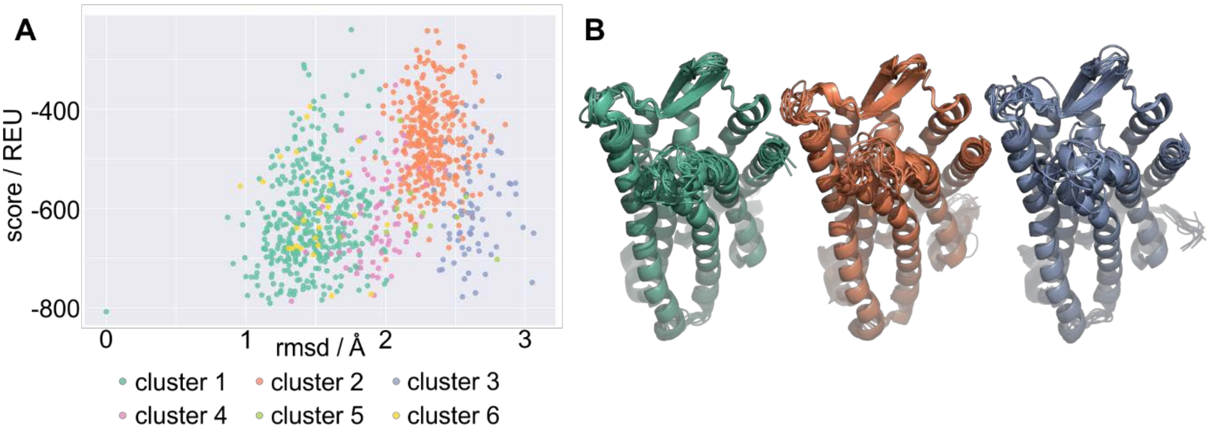

Figure S2: *Homology Models of GPR1 in the ligand-free state.* **A)** *The resulting models were clustered and analyzed regarding their total score and their rmsd to the best scoring models.* **B)** *The 10 best scoring models from cluster 1 (left), cluster 2 (center) and cluster 3 (right) were used to dock the ligand using Rosetta FlexPepDock.*

Table S1: *Alignment used for homology modeling of GPR1. Conserved x.50 residues are highlighted in gray.*

	1 … 1.50 (N) … 50
**GPR1**	- - - - - - - E E K V Q L G V V H W V S L V L Y C L A F V L G I P G **N** A I V I W F T G F K W K - K -
**CCR9**	- - - - - - - - - - - - - - - R Q F A S H F L P P L Y W L V F I V G A L G **N** S L V I L V Y W Y C A R A K -
**APJR**	- - - - - - - - C E Y T D W K S S G A L I P A I Y M L V F L L G T T G **N** G L V L W T V F R S S R E K R
**C5aR1**	- - - - - - - - - - S N T L R V P D I L A L V I F A V V F L V G V L G **N** A L V V W V T A F E A K - R -
**CXCR4**	- - - - - P C F R E E N A N F N K I F L P T I Y S I I F L T G I V G **N** G L V I L V M G Y Q K K L R -
**AT1R**	I L N S S D C P K A G R H N Y I F V M I P T L Y S I I F V V G I F G **N** S L V V I V I Y F Y M K L K -
	2.50 (D) … 100
**GPR1**	T V T T L W F L N L A I A **D** F I F L L F L P L Y I S Y V A M - N F H W P F G I W L C K A N S F T A Q
**CCR9**	T A T D M F L L N L A I A **D** L L F L V T L P F W A I A - - - - - - - - - - - - - T F M C K V V N S M Y K
**APJR**	R S A D I F I A S L A V A **D** L T F V V T L P L W A T Y T Y R - D Y D W P F G T F F C K L S S Y L I F
**C5aR1**	T I N A I W F L N L A V A **D** F L S C L A L P I L F T S I V Q - H H H W P F G G A A C S I L P S L I L
**CXCR4**	S M T D K Y R L H L S V A **D** L L F V I T L P F W A V D A V A - - - N W Y F G N F L C K A V H V I Y T
**AT1R**	T V A S V F L L N L A L A **D** L C F L L T L P L W A V Y T A M - E Y R W P F G N Y L C K I A S A S V S
	3.50 (H/R) … 4.50 (W) … 150
**GPR1**	L N M F A S V F F L T V I S L D **H** Y I H L I H P V L S H R H R - - T L K N S L I V I I F I **W** L L A S
**CCR9**	M N F Y S C V L L I M C I C V D **R** Y I A I A Q A M R A H T W R E K R L L Y S K M V C F T I **W** V L A A
**APJR**	V N M Y A S A F C L T G L S F D **R** Y L A I V R P V A N A R L R - - L R V S G A V A T A V L **W** V L A A
**C5aR1**	L N M Y A S I L L L A T I S A D **R** F L L V F K P I W C Q N F R - - G A G L A W I A C A V A **W** G L A L
**CXCR4**	V N L Y S S V W I L A F I S L D **R** Y L A I V H A T N S Q R P R - - K L L A E K V V Y V G V **W** I P A L
**AT1R**	F N L Y A S V F L L T C L S I D **R** Y L A I V H P - - - - - - - - - - T M L V A K V T C I I I **W** L L A G
	200
**GPR1**	L I G G P A L Y F R D T V E F N - - N H T L C Y N N F Q - K H D P D L T L I R H H V L T W V K F I I
**CCR9**	A L C I P E I L Y - - - - - - - - - - - - - - C T - - - - - - - - - - - - - - T K L K S A V L A L K V I L
**APJR**	L L A M P V M V L R T T G D L E N T N K V Q C Y M D Y S M V A T V S S E W A W E V G L G V S S T T V
**C5aR1**	L L T I P S F L Y R V V R E E Y F P P K V L C G V - - - - - - - D Y S H D K R R E R A V A I V R L V L
**CXCR4**	L L T I P D F I F A N V S E A D - - D R Y I C D R F Y P - - - - - - - - N D L W V V V F Q F Q H I M V
**AT1R**	L A S L P A I I H R N V F F I E N T N I T V C A F H Y E - - - - - - - - - S T L P I G L G L T K N I L
	5.50 (P) … 6.50 (P) 300
**GPR1**	G Y L F **P** L L T M S I C Y L C L I F K V K K R S I L I S S R H F W T I L V V V V A F V V C W T **P** Y H
**CCR9**	G F F L **P** F V V M A C C Y T I I I H T L I Q A K K S S K H K A L K A T I T V L T V F V L S Q F **P** Y N
**APJR**	G F V V **P** F T I M L T C Y F F I A Q T I A - - - - - - - R R R L L S I I V V L V V T F A L C K M **P** Y H
**C5aR1**	G F L W **P** L L T L T I C Y T F I L L R T W S R R - - - S T K T L K V V V A V V A S F F I F W L **P** Y Q
**CXCR4**	G L I L **P** G I V I L S C Y C I I I S K L S H S K G H Q K R K A L K T T V I L I L A F F A C W L **P** Y Y
**AT1R**	G F L F **P** F L I I L T S Y T L I W K A L - - - - - - - - N D D I F K I I M A I V L F F F F S W I **P** H Q
	7.50 (P) 350
**GPR1**	L F S I W E L T I - - - H H N - S Y S - - - - H H V M Q A G I P L S T G L A F L N S C L N **P** I L Y V
**CCR9**	C I L L V Q T I D A Y A M F I - - S N - C A V S T A I D I C F Q V T Q A I A F F H S C L N **P** V L Y V
**APJR**	L V K T L Y M L G S L L H - - - - W P - C D F D L F L M N I F P Y C T C I S Y V N S C L N **P** F L Y A
**C5aR1**	V T G I M M S F L - - - E P S S P T F - - - - - L L L K K L D S L C V S F A Y I N C C I N **P** I I Y V
**CXCR4**	I G I S I D S F I L L E I I K - - Q G - C E F E N T V H K W I S I T E A L A F F H C C L N **P** I L Y A
**AT1R**	I F T F L D V L I Q L G I I - - - R D - C R I A D I V D T A M P I T I C I A Y F N N C L N **P** L F Y G
**GPR1**	L I S K K F Q A R F R S S V A E I L K - - - - - - - -
**CCR9**	F V G E R F R R D L V K T L K N L G A I S Q A A A H H
**APJR**	F F D P R F R Q A C T S M L L M G Q S R - - - - - - -
**C5aR1**	V A G Q G F - - - - - - K S L P S L L R N V L T E E S -
**CXCR4**	F L G A K F K T S A Q H A L T S - - - - - - - - - - -
**AT1R**	F -

Using FlexPepDock *ab initio*, 25,000 models of the docked complex were produced. The best 2000 models by total score were clustered based on Cα RMSD and analyzed regarding their interface score ΔG separate (Figure S3). Taking total and interface score into account, models from cluster 2 seemed to be the best fit. Models from cluster 1 are better judging from the total score alone, but residue $F^{4.79}$ faces TM4 and 5 and is not available to interact with the ligand (Figure S3B). This is in contrast to our experimental data showing that $F^{4.79}$ is a highly important residue for ligand binding. Models from cluster 2 better account for this fact, which prompted us to select these models for refinement using Rosetta FastRelax.

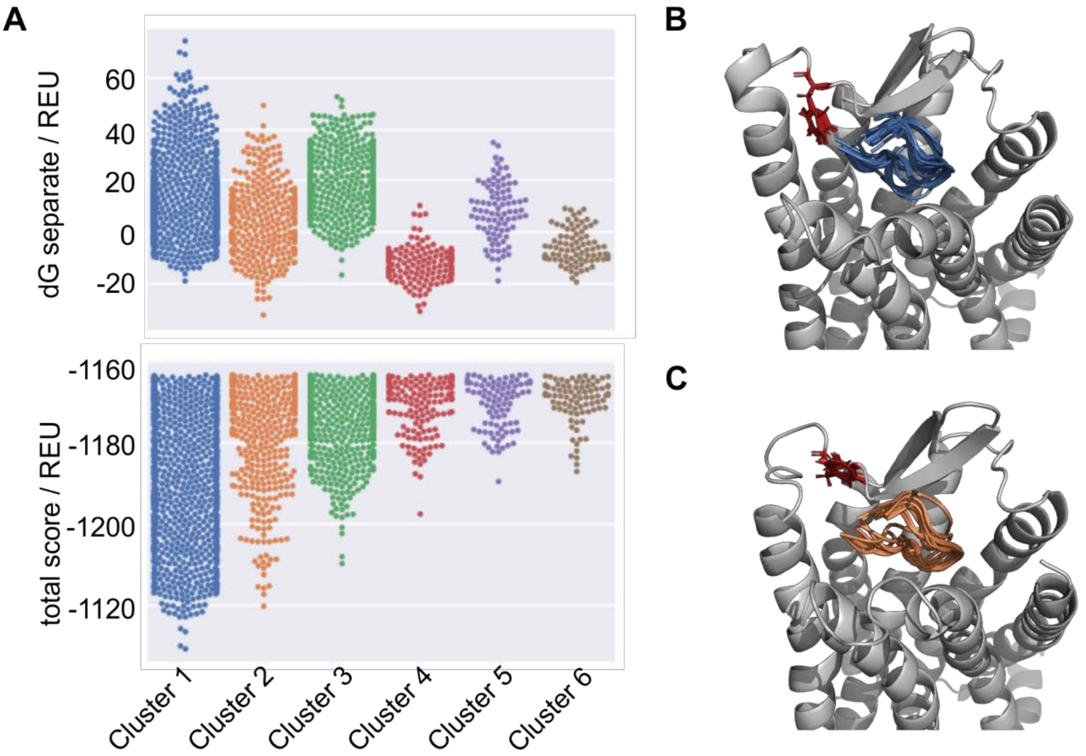

Figure S3: *Analysis of docked models after the first docking step with Rosetta FlexPepDock.* **A)** *Energetic analysis of models regarding interface score (top) and total score (bottom). Clusters are ordered by decreasing size.* **B)** *Best scoring 20 models from cluster 1 with the ligand shown in blue and residue $F^{4.79}$ in red.* **C)** *Best scoring 20 models from cluster 2 with the ligand shown in orange and $F^{4.79}$ in red.*

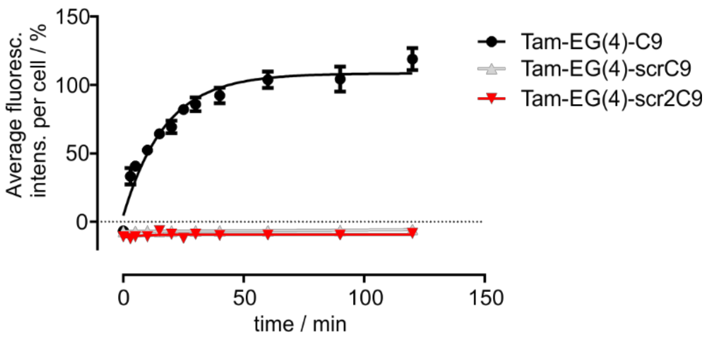

Figure S4: *HEK293 cells stably expressing CMKLR1 internalize Tam-EG(4)-chemerin-9, but not the scrambled peptides Tam-EG(4)-scrC9 and Tam-EG(4)-scr2C9. Cells were stimulated with 1 µM of the respective peptide; data points represent mean ± SEM*

Analytical Characterization of Peptides and Proteins

Table 2: Analytical Characterization of chemerin proteins and derived peptides. *All proteins and peptides displayed a purity of at least 95 % as determined by the absorption at 220 nm in RP-HPLC. Mass spectrometry was performed on a Bruker Ultraflex III in linear mode for the proteins ChemS157 and ChemF156, and in reflector mode for all peptides.*

	**Sequence**	**M_{calc} / Da**	**M_{obs}**	**t_R / %B**	**t_R / %B**
ChemS157	Chemerin$^{21\text{-}157}$	18392	18393	38.9^{c,2}	-
ChemF156	Chemerin$^{21\text{-}156}$	18305	18306	39.2^{c,2}	-
Chem$^{139\text{-}157}$	QRAGEDPHSFYFPGQFAFS	2186.9	2187.9	40.3^{a,1}	31.8^{b,1}
Chem$^{139\text{-}156}$	QRAGEDPHSFYFPGQFAF	2099.9	2100.9	41.8^{a,1}	33.3^{b,1}
Chem$^{149\text{-}157}$	YFPGQFAFS	1062.5	1063.6	41.8^{a,1}	30.2^{c,1}
Chem$^{150\text{-}157}$	FPGQFAFS	899.4	900.4	34.9^{a,1}	10.2^{b,1}
Chem$^{151\text{-}157}$	PGQFAFS	752.3	753.3	30.0^{a,1}	26.1^{b,1}
Chem$^{149\text{-}156}$	YFPGQFAF	975.5	976.5	44.8^{a,1}	34.1^{b,1}
Chem$^{149\text{-}155}$	YFPGQFA	828.4	829.4	31.8^{a,2}	25.8^{b,2}
scrC9	GYFPFQASF	1062.5	1063.5	40.1^{a,1}	35.6^{c,1}
scr2C9	QFYSFFPAG	1062.5	1063.5	38.1^{a,1}	33.5^{b,1}
[N-C]-c(chemerin-9	[YFPGQFAFS]	1044.5	1045.5	43.5^{a,1}	36.6^{b,1}
[4-9]-c(chemerin-9)	YFP[D-Hcys-QFAFC]	1136.5	1137.5	45.6^{a,1}	34.1^{b,1}
[L8]-chemerin-9	YFPGQFALS	1028.5	1029.5	34.9^{a,1}	26.8^{c,1}
Tam-EG(4)-chemerin-9	Tam-EG(4)-YFPGQFAFS	1721.7	1722.7	51.0^{a,1}	40.5^{c,1}
Tam-EG(4)-[L8]-chemerin-9	Tam-EG(4)-YFPGQFALS	1687.8	1688.9	44.8^{a,1}	39.6^{c,1}

Tam-EG(4)-scrC9	Tam-EG(4)-GYFPFQASF	1721.8	1722.7	46.8[a,1]	42.3[b,1]
Tam-EG(4)-scr2C9	Tam-EG(4)- QFYSFFPAG	1721.8	1722.8	46.2[a,1]	41.8[b,1]

t_R: Elution in RP-HPLC with a linear gradient of [1]20-70%B in A over 40 min or [2]10-60%B in A over 40 min with a flow rate of [a]1 mL/min on a Jupiter 4 µm Proteo 90Å C_{12} column, [b]1.55 mL/min on a Kinetex 5 µm biphenyl 100 Å column, or [c]1.55 mL/min on an Aeris 3.6 µm 100 Å XB-C18 column.

Protocol Capture Molecular Modeling

GPR1 models in the ligand-free state were generated using RosettaCM. Fusion proteins, salt and water atoms were removed from the template structures prior to modeling. A span file was generated using the Octopus web server (http://octopus.cbr.su.se/) with the truncated GPR1 sequence that was used for homology modeling.

Sequence to Generate GPR1 Homology Models-----------------------

```
GVVHWVSLVLYCLAFVLGIPGNAIVIWFTGFKWKKTVTTLWFLNLAIADFIFLLFLPL
YISYVAMNFHWPFGIWLCKANSFTAQLNMFASVFFLTVISLDHYIHLIHPVLSHRHRT
LKNSLIVIIFIWLLASLIGGPALYFRDTVEFNNHTLCYNNFQKHDPDLTLIRHHVLTW
VKFIIGYLFPLLTMSICYLCLIFKVKKRSILISSRHFWTILVVVVAFVVCWTPYHLFS
IWELTIHHNSYSHHVMQAGIPLSTGLAFLNSCLNPILYVLISKKFQARFRSSVAEILK
```

RosettaScripts Options for Homology Modeling:---------------------

```
# i/o
-in:file:fasta
/home/fischet/Documents/GPR1/1_rosetta_apo/rosetta_cm/targ
et.fasta
-nstruct 100
-parser:protocol
/home/fischet/Documents/GPR1/1_rosetta_apo/rosetta_cm/rose
tta_cm.xml
-out:pdb_gz
-out:path:all
/home/fischet/Documents/GPR1/1_rosetta_apo/rosetta_cm/outp
ut/

# membrane options
-in:file:spanfile
/home/fischet/Documents/GPR1/1_rosetta_apo/rosetta_cm/span
.txt
-membrane:no_interpolate_Mpair
-membrane:Menv_penalties
-rg_reweight .1

# relax options
-default_max_cycles 200
-relax:min_type lbfgs_armijo_nonmonotone
-relax:minimize_bond_angles
-relax:minimize_bond_lengths
-relax:jump_move true
-score:weights
/dors/meilerlab/home/fischet/Documents/weights/stage3_rlx_
membrane.wts
-use_bicubic_interpolation
-hybridize:stage1_probability 1.0
```

```
-sog_upper_bound 15

# reduce memory footprint
-chemical:exclude_patches LowerDNA  UpperDNA
Cterm_amidation SpecialRotamer VirtualBB ShoveBB
VirtualDNAPhosphate VirtualNTerm CTermConnect sc_orbitals
pro_hydroxylated_case1 pro_hydroxylated_case2
ser_phosphorylated thr_phosphorylated  tyr_phosphorylated
tyr_sulfated lys_dimethylated lys_monomethylated
lys_trimethylated lys_acetylated glu_carboxylated
cys_acetylated tyr_diiodinated N_acetylated
C_methylamidated MethylatedProteinCterm

-linmem_ig 10

# run multiple processes to produce output for one file
-multiple_processes_writing_to_one_directory
```

RosettaScripts Protocol for Homology Modeling---------------------------------------

```
<ROSETTASCRIPTS>
    <TASKOPERATIONS>
    </TASKOPERATIONS>
    <SCOREFXNS>
        <ScoreFunction name="stage1"
weights="/dors/meilerlab/home/fischet/Documents/weights/st
age1_membrane.wts" symmetric="0">
            <Reweight scoretype="atom_pair_constraint"
weight="1"/>
        </ScoreFunction>
        <ScoreFunction name="stage2"
weights="/dors/meilerlab/home/fischet/Documents/weights/st
age2_membrane.wts" symmetric="0">
            <Reweight scoretype="atom_pair_constraint"
weight="0.5"/>
        </ScoreFunction>
        <ScoreFunction name="fullatom"
weights="/dors/meilerlab/home/fischet/Documents/weights/st
age3_rlx_membrane.wts" symmetric="0">
            <Reweight scoretype="atom_pair_constraint"
weight="0.5"/>
        </ScoreFunction>
        <ScoreFunction name="membrane"
weights="membrane_highres_Menv_smooth" symmetric="0">
            <Reweight scoretype="cart_bonded"
weight="0.5"/>
            <Reweight scoretype="pro_close" weight="0"/>
        </ScoreFunction>
    </SCOREFXNS>
    <FILTERS>
```

```
    </FILTERS>
    <MOVERS>
        <Hybridize name="hybridize"
stage1_scorefxn="stage1" stage2_scorefxn="stage2"
fa_scorefxn="fullatom" batch="1"
stage1_increase_cycles="1.0" stage2_increase_cycles="1.0"
linmin_only="1" realign_domains="0"
disulf_file="/home/fischet/Documents/GPR1/1_rosetta_apo/ro
setta_cm/disulf.txt">
            <Template
pdb="/home/fischet/Documents/GPR1/1_rosetta_apo/rosetta_cm
/5lwe__thread.pdb" cst_file="AUTO" weight="1.000" />
            <Template
pdb="/home/fischet/Documents/GPR1/1_rosetta_apo/rosetta_cm
/5vbl__thread.pdb" cst_file="AUTO" weight="1.000" />
            <Template
pdb="/home/fischet/Documents/GPR1/1_rosetta_apo/rosetta_cm
/6c1r__thread.pdb" cst_file="AUTO" weight="1.000" />
            <Template
pdb="/home/fischet/Documents/GPR1/1_rosetta_apo/rosetta_cm
/3odu__thread.pdb" cst_file="AUTO" weight="1.000" />
            <Template
pdb="/home/fischet/Documents/GPR1/1_rosetta_apo/rosetta_cm
/4zud__thread.pdb" cst_file="AUTO" weight="1.000" />
        </Hybridize>
        <ClearConstraintsMover name="clearconstraints"/>
    </MOVERS>
    <APPLY_TO_POSE>
    </APPLY_TO_POSE>
    <PROTOCOLS>
        <Add mover="hybridize"/>
        <Add mover="clearconstraints"/>
    </PROTOCOLS>
    <OUTPUT scorefxn="membrane"/>
</ROSETTASCRIPTS>
```

Docking Chemerin-9 to GPR1

After model selection, chemerin-9 was docked into the 10 best scoring GPR1 homology models from each of the three largest clusters. The peptide was placed in the putative binding pocket using pymol. Rosetta FlexPepDock was used in *ab initio* mode to generate 25,000 models using the following options and constraints.

Constraints for Rosetta FlexPepDock----------------------------

```
SiteConstraint CB 62A C FLAT_HARMONIC 5.0 1.0 2.0
SiteConstraint CB 67A C FLAT_HARMONIC 5.0 1.0 2.0
SiteConstraint CB 154A C FLAT_HARMONIC 5.0 1.0 2.0
SiteConstraint CB 157A C FLAT_HARMONIC 5.0 1.0 2.0
SiteConstraint CB 235A C FLAT_HARMONIC 5.0 1.0 2.0
```

```
AtomPair CB 145 CG 298 FLAT_HARMONIC 4 1 2.5
AtomPair CG 147 CG 298 FLAT_HARMONIC 4 1 2.5

AtomPair CA 291 CA 299 FLAT_HARMONIC 5 1 3
```

Options for Rosetta FlexPepDock-------------------

```
-database /dors/meilerlab/apps/rosetta/rosetta-
3.9/main/database/

-in:file:l ./pdb.lst
-in:file:spanfile ./input_files/span.txt

-out:pdb_gz
-out:path:all output/
-out:file:scorefile dock1.sc

-lowres_abinitio
#-extend_peptide

-ex1
-ex2aro
-use_input_sc

-frag3 ./input_files/chem9_frags.200.3mers
-frag5 ./input_files/chem9_frags.200.5mers

-constraints:cst_file input_files/chem9.cst
-constraints:cst_weight 10
-constraints:cst_fa_weight 10

-score:weights membrane_highres_Menv_smooth
#-multiple_processes_writing_to_one_directory
```

Refinement of Models Using Rosetta FastRelax

The best scoring models from cluster 2 were refined using Rosetta FastRelax without applying any constraints. This step was restricted to the extracellular part of GPR1 and the ligand.

Options for Rosetta Relax--------------------------------

```
-database /dors/meilerlab/apps/rosetta/rosetta-
3.9/main/database/

-in:file:l ./pdb.lst

-use_input_sc                # Include rotamers from the
input structure
#-nstruct 5                   # Generate 5 models
-out:pdb_gz
-out:path:all output/
```

```
-out:file:scorefile relax.sc
-ex1
```

RosettaScripts Protocol for FastRelax-------------------------------

```
<ROSETTASCRIPTS>
     <SCOREFXNS>
             <ScoreFunction name="ref2015"
weights="ref2015.wts" >
          </ScoreFunction>
     </SCOREFXNS>
     <RESIDUE_SELECTORS>
          <Index name="loops" resnums="1-10,56-86,136-
180,231-255,291-299"/>
     </RESIDUE_SELECTORS>
     <MOVE_MAP_FACTORIES>
          <MoveMapFactory name="movemap_L1" bb="0"
chi="0">
               <Backbone residue_selector="loops" />
               <Chi residue_selector="loops" />
          </MoveMapFactory>
     </MOVE_MAP_FACTORIES>
     <TASKOPERATIONS>
     </TASKOPERATIONS>
     <FILTERS>
     </FILTERS>
     <MOVERS>
          <FastRelax name="relax_loops"
movemap_factory="movemap_L1" scorefxn="ref2015">
          </FastRelax>
          <InterfaceAnalyzerMover name="analyze"
scorefxn="ref2015" packstat="0" pack_input="0"
pack_separated="1" fixedchains="A" />
     </MOVERS>
     <APPLY_TO_POSE>
     </APPLY_TO_POSE>
     <PROTOCOLS>
          <Add mover="relax_loops"/>
          <Add mover="analyze"/>
     </PROTOCOLS>
     <OUTPUT scorefxn="ref2015" />
</ROSETTASCRIPTS>
```
